# Vertical canopy gradient shaping the stratification of leaf‐chewer–parasitoid interactions in a temperate forest

**DOI:** 10.1002/ece3.4194

**Published:** 2018-06-27

**Authors:** Martin Šigut, Hana Šigutová, Jan Šipoš, Petr Pyszko, Nela Kotásková, Pavel Drozd

**Affiliations:** ^1^ Department of Biology and Ecology University of Ostrava Ostrava Czech Republic; ^2^ Institute of Environmental Technologies University of Ostrava Ostrava Czech Republic; ^3^ Department of Vegetation Ecology Institute of Botany CAS Brno Czech Republic; ^4^ Department of Zoology, Fisheries, Hydrobiology and Apiculture Mendel University in Brno Brno Czech Republic

**Keywords:** herbivore–parasitoid interactions, host specificity, parasitism rate, quantitative food webs, temperate forest canopy, vertical stratification

## Abstract

Knowledge about herbivores and their parasitoids in forest canopies remains limited, despite their diversity and ecological importance. Thus, it is important to understand the factors that shape the herbivore–parasitoid community structure, particularly the effect of vertical gradient. We investigated a quantitative community dataset of exposed and semiconcealed leaf‐chewing larvae and their parasitoids along a vertical canopy gradient in a temperate forest. We sampled target insects using an elevated work platform in a 0.2 ha broadleaf deciduous forest plot in the Czech Republic. We analyzed the effect of vertical position among three canopy levels (first [lowest], second [middle], and third [highest]) and tree species on community descriptors (density, diversity, and parasitism rate) and food web structure. We also analyzed vertical patterns in density and parasitism rate between exposed and semiconcealed hosts, and the vertical preference of the most abundant parasitoid taxa in relation to their host specificity. Tree species was an important determinant of all community descriptors and food web structure. Insect density and diversity varied with the vertical gradient, but was only significant for hosts. Both host guilds were most abundant in the second level, but only the density of exposed hosts declined in the third level. Parasitism rate decreased from the first to third level. The overall parasitism rate did not differ between guilds, but semiconcealed hosts suffered lower parasitism in the third level. Less host‐specific taxa (Ichneumonidae, Braconidae) operated more frequently lower in the canopy, whereas more host‐specific Tachinidae followed their host distribution. The most host‐specific Chalcidoidea preferred the third level. Vertical stratification of insect density, diversity, and parasitism rate was most pronounced in the tallest tree species. Therefore, our study contradicts the general paradigm of weak arthropod stratification in temperate forest canopies. However, in the network structure, vertical variation might be superseded by variation among tree species.

## 1 INTRODUCTION

Herbivores and their parasitoids represent a major component of global insect diversity (Lewinsohn & Roslin, 2008; Price, 2002). Being one of the fundamental forces determining population dynamics and community structure in terrestrial ecosystems, parasitoids regulate herbivorous insect populations influencing host–plant use and promoting species diversification (Lill, Marquis, & Ricklefs, 2002; Stireman & Singer, 2003). Parasitoids operate at high trophic level; moreover, they are often remarkably specialized, but their autecology is poorly understood. Therefore, parasitoids are a highly threatened group that is extremely vulnerable to extinction (Shaw & Hochberg, 2001; Smith, Wood, Janzen, Hallwachs, & Hebert, 2007). In temperate and tropical forests, the major source of insect diversity is the forest canopy (Novotny & Basset, 2005; Stork, 1988). Many arthropod species in temperate deciduous forests depend on canopy habitats, and approximately half of them are predators or parasitoids (Moran & Southwood, 1982; Ulyshen, 2011). Thus, studying herbivore–parasitoid communities in forest canopies could enhance our understanding of their structure and diversity (Stireman, Cerretti, Whitmore, Hardersen, & Gianelle, 2012). Moreover, investigating differences in ecological communities along habitat gradients could help us to recognize the factors that determine community structure (Morris, Sinclair, & Burwell, 2015).

Many studies have investigated the vertical stratification of arthropods (e.g., Basset, Horlyck, & Wright, 2003; Basset et al., 2007; Stork, Adis, & Didham, 1997). However, these studies are significantly biased toward the tropics (Basset, Hammond, Barrios, Holloway, & Miller, 2003; Lowman, Taylor, & Block, 1993; Ulyshen, 2011), perhaps owing to the belief that temperate deciduous forests have less pronounced vertical gradients in microclimate and biotic factors, and support a smaller proportion of highly specialized canopy‐restricted species (Basset, Hammond, et al., 2003; Lowman et al., 1993; Ulyshen, 2011). Therefore, the general paradigm is that the vertical stratification of herbivores in temperate forest canopies is weak (Basset, Hammond, et al., 2003; Fowler, 1985). However, temperate canopies have not been well studied, even though they exhibit changes in vertical foliage complexity, quality, chemistry, and toughness, as well as microclimate (Parker, 1995; Ulyshen, 2011). Therefore, we might expect the stratification of herbivores and associated parasitoids to exist in temperate forests. However, studies of parasitoid stratification are also scarce and are often limited to surveys of adults (e.g., Compton, Ellwood, Davis, & Welch, 2000; Pucci, 2008; Stireman et al., 2012; Vance, Smith, Malcolm, Huber, & Bellocq, 2007), restricting our understanding of trophic links. Consequently, knowledge of the stratification of parasitoids and parasitism rates along a vertical gradient in a temperate forest remains limited.

Recently, there has been a shift from simply documenting the incidence of interactions among herbivores and their parasitoids toward establishing quantitative food webs, which provide further insights into the processes shaping herbivorous insect communities and their structure (Memmott & Godfray, 1994; van Veen, Morris, & Godfray, 2006). However, only three studies have investigated the vertical stratification of host–parasitoid food webs (Chaij, Devoto, Oleiro, Chaneton, & Mazía, 2016; Morris et al., 2015; Paniagua, Medianero, & Lewis, 2009). Therefore, more studies on the quantitative food webs of herbivorous insect–parasitoid communities in forest canopies are needed to understand differences between forest types and the particularities of various herbivorous guilds. Furthermore, obtaining selective samples along the whole vertical transect of the canopy could provide much more detailed and robust information.

Here, we investigated a quantitative community dataset of exposed and semiconcealed leaf‐chewing larvae and their parasitoids along a vertical gradient in the forest canopy. In a 0.2 ha plot of temperate floodplain forest, we used an elevated truck‐mounted work platform to access the canopy up to 40 m, enabling us to collect specimens at stratified canopy positions. We tested for the differences among three separate vertical canopy levels: first (closest to the forest floor), second (in the middle), and third (the uppermost layer of a given tree). We tested four hypotheses.

Hypothesis (i): Insect density, diversity, and parasitism rate change along a vertical canopy gradient, differing among tree species and changing within a season. Specifically, we expect density, diversity, and parasitism rates to decrease from the first toward the third canopy level, due to harsher abiotic conditions and lower foliage quality toward the upper canopy (Hirao, Murakami, & Kashizaki, 2009; Le Corff & Marquis, 1999). Moreover, in temperate forests, the understory contributes to lepidopteran diversity more than the canopy (Hirao et al., 2009; Le Corff & Marquis, 1999), with canopy and understory assemblages potentially sharing many species (Hirao et al., 2009). Therefore, we also expect an increased involvement of understory species in the first canopy level, leading to an increase in their density and diversity, and, consequently, parasitoid density and diversity. Furthermore, we expect insect species composition to differ among tree species, due to the bottom‐up effects of plant chemistry and herbivore host specificity (Murakami, Hirao, & Ichie, 2007; Volf et al., 2017), and along a vertical canopy gradient due to the changing biotic and abiotic factors mentioned above.

Hypothesis (ii): Vertical patterns in density and parasitism differ between exposed and semiconcealed hosts. We expect both groups to be more parasitized lower in the canopy, but we expect semiconcealed hosts to be more abundant higher in the canopy, in accordance with the hypothesis that the harsh upper canopy environment favors concealed or semiconcealed feeders (Ribeiro & Basset, 2007). Moreover, in tropical forests, there are differences in parasitoid community composition between exposed and semiconcealed feeders (Hrcek, Miller, Whitfield, Shima, & Novotny, 2013), and here, we expect a similar pattern.

Hypothesis (iii): Vertical distribution patterns of the most abundant parasitoid groups differ in relation to their host specificity. We expect the distribution of parasitoid specialists to be determined by the distribution of their hosts, whereas we expect the distribution of generalists to be constrained by environmental differences among strata (Basset, Hammond, et al., 2003).

Hypothesis (iv): Quantitative food web structure changes along a vertical canopy gradient and differs among tree species. In temperate forests, leaf miner–parasitoid food web complexity decreases with canopy height, as a reflection of decreasing parasitism rate (Chaij et al., 2016). We expect a similar pattern, reflecting vertical changes in quantitative network indices. Specifically, we expect weighted connectance, linkage density, interaction evenness, specialization, generality, vulnerability, and nestedness to decrease from the first to third canopy level. In comparison, we expect modularity and number of compartments to increase from the first toward the third canopy level.

Our results are expected to provide new insights into whether temperate forest canopies reflect the general paradigm of weak arthropod stratification detected in tropical forests.

## 2 MATERIALS AND METHODS

### 2.1 Field sampling and insect rearing

External leaf‐chewing Lepidoptera and Hymenoptera larvae (hereafter referred to as hosts) were sampled from trees with a diameter at breast height >5 cm in a 0.2 ha broadleaf deciduous forest plot in Lanžhot, Czech Republic (48°41′22.866″N, 16°56′41.071″E). We used an elevated truck‐mounted work platform (GENIE Z‐135/70 JRT; Genie Industries, Redmond, WA, USA) with a 40‐m arm to access the entire vertical gradient. Branches were sampled individually by a two‐man crew, starting from the base and working toward the top, using 1‐m^2^ beating sheets and exhausters. We manually collected any remaining larvae. This method enabled approximately 90% of the canopy foliage to be sampled. Leaf number for each branch was estimated visually, independently by two persons, and the mean value of both estimates was used. Subsequently, a subsample of leaves from each tree was photographed in a 50 × 50‐cm frame with a white background; their area was calculated in ImageJ 1.48v (Rasband, 2014), and the results were used to convert leaf number to leaf area per branch. Total tree height and the vertical position of individual branches were measured by a digital laser distance meter HECHT^®^ 2006 (Hecht Motors Inc., Czech Republic). For our study, the branches of each tree were merged together to create three separate vertical levels (first, closest to the forest floor; second, in the middle; and third, the uppermost layer of a given tree), where height depended on the height and structure of individual trees, with each level covering an approximately equal leaf area. It is important to note that, in our study, the term “third canopy level” should not be confused with “upper canopy” (i.e., uppermost leaf layer and the volume few meters below; Basset, Hammond, et al., 2003). Upper canopy offering harsh abiotic conditions was sensu stricto present only in the highest trees; thus, we strictly distinguish between the terms “third level” and “upper canopy” in this study.

Sampling was conducted from May to August 2013 and 2014, in approximately 1‐month intervals, and in May 2015, resulting in 10 sampling events, together fully covering the whole seasonal gradient. During each event, at least one individual of each of the most abundant tree species (*Acer campestre*,* Carpinus betulus*,* Fraxinus angustifolia*, and *Quercus cerris*) was sampled to capture host–plant diversity. In particular, for tree species that represented the smallest leaf area, we sampled more than one individual per sampling event to compensate for different sampling effort among tree species. For the least abundant tree species (*Tilia cordata* and *Fraxinus excelsior*), we were not able to cover all sampling events; therefore, these species were only sampled in the peak of the season (May, June) to obtain most of the specimens. For the subsequent analyses, the data for *F. angustifolia* and *F. excelsior* were merged because of their similar architectural, mechanical, and chemical traits. The sampled larvae were morphotyped, photographed, and transferred to plastic containers (one larva per container), where they were provided with host–plant material from the plant species they were collected from. In the laboratory, larvae were provided with new fresh host–plant material when necessary and reared until either adults or parasitoids emerged, or they died (Basset, Novotny, Miller, & Pyle, 2000).

### 2.2 Insect identification

Host larvae were assigned to morphospecies before rearing. All larvae and reared adult hosts were identified by N. K. and P. P., or by collaborating taxonomists, by combining morphology, larval stage photographs, and morphotype assignments. Adult identifications were based on the available literature (Supporting Information Table S1) or were confirmed by dissection of the genitalia. Specimens for which we could not confidently assign morphotypes (remnants of parasitized hosts, specimens in bad condition, or those not verified by rearing; 418 hosts, 4.0%) underwent DNA barcoding of the cytochrome oxidase subunit I (COI) gene region at the Canadian Centre for DNA Barcoding (CCDB, University of Guelph), using standardized protocols (deWaard, Ivanova, Hajibabaei, & Hebert, 2008; Ivanova, deWaard, & Hebert, 2006). According to field observations, hosts were assigned to a semiconcealed (leaf rollers, tiers, shelter builders) or exposed (freely foraging) feeding guild. Species that did not feed on the host–plant material they were provided with and whose host plant was not verified in the literature were considered transient (Basset et al., 2000) and were excluded from further analyses (187 host individuals, 1.8%). For further species‐level analyses, we used 177 successfully identified leaf chewer species; for other analyses, all individuals (un/identified) were used.

All reared parasitoids (adults or pupae), or unreared specimens dissected from dead host larvae and pupae, were sorted, morphotyped, and identified to family level by M. Š., using taxonomic keys and online databases (Supporting Information Table S1). Based on morphotyping, a selection of parasitoid specimens (735 individuals, 58.5%) was DNA barcoded: 694 at CCDB, following the stated protocols, and 41 at the University of Ostrava, Czech Republic (sequencing performed by Macrogen Inc., Seoul, South Korea) using standardized protocols (Hrcek, Miller, Quicke, & Smith, 2011). Generated sequences were uploaded to BOLD (http://www.boldsystems.org/) and assigned barcode index numbers (BINs, i.e., putative species). In total, 122 parasitoid BINs were found by DNA barcoding. For further species‐level analyses, we treated these BINs as species (Ratnasingham & Hebert, 2013). In addition, 21 distinct morphospecies, which were not successfully barcoded, were also considered as species, altogether resulting in 143 putative parasitoid species. Voucher specimens were deposited at the University of Ostrava and Zoologische Staatssammlung Munich, Germany.

### 2.3 Data analysis

#### 2.3.1 Insect density

All analyses were performed using the statistical program R (R Development Core Team, 2017).

First, we evaluated whether host and parasitoid density differed among tree species. Therefore, we fitted two (one for hosts and one for parasitoids) generalized linear models (GLMs) with Poisson distribution of error variance and log link function. To adjust for the effect of leaf area, we converted the abundance to be a rate per unit of leaf area. For this purpose, the relationship between mean abundance and the linear predictor of the model was offset by a common logarithm of the leaf area; therefore, abundance was defined as density. Offset is a predictor variable for which the coefficient is fixed at 1. To compensate for the confounding effect of temporal variability, we used season (i.e., month of particular sampling event) as a covariate with third‐degree polynomial. To test for the effect of canopy level and season on the density of hosts and parasitoids, we fitted two linear mixed‐effect models (LMMs) in the package nlme (Pinheiro, Bates, DebRoy, & Sarkar, 2017). Based on the results of testing for the effect of tree species, tree species was set as a random effect. The dependent variable was log transformed to ensure that residuals had a normal distribution. Season (i.e., month of particular sampling event) was set as a covariate with third‐degree polynomial to remove (or parcel out) all variability associated with it. Dependent variables were weighted by leaf area, as in the case of the GLM. In each LMM, we used two explanatory variables (canopy level and season) and the interaction between canopy level and tree species. The significance of each explanatory variable was tested using analysis of deviance with *F* statistics.

#### 2.3.2 Insect diversity

First, we tested for the effect of tree species on host and parasitoid diversity. Therefore, we fitted two GLMs, as in the case of density, with a single exception. Except for leaf area, total abundance was used as the other covariate to compensate for sampling effort. To test for the effect of canopy level and season on the number of host and parasitoid species, we used a similar LMM to that used for insect density, with the tree species being set as a random effect (based on the results of the GLM). However, the total abundance was used as the other covariate to compensate for sampling effort. Furthermore, patterns in host and parasitoid diversity were examined among canopy levels and tree species, using individual‐based rarefaction curves. To construct the curves, we used interpolation and extrapolation approaches on data pooled across all sampling events. Extrapolation was only used for tree species and canopy levels with a low number of collected individuals. The endpoint of each rarefaction curve was specified as the smallest sample size among all measurements. Confidence intervals and species diversity were estimated by the bootstrap resampling method based on 500 replications. For each diversity measure, we used the iNEXT package (Hsieh, Ma, & Chao, 2016), computing species diversity for rarefied and extrapolated samples with respect to sample size (Chao et al., 2014).

#### 2.3.3 Parasitism rate

The parasitism rate was quantified as the proportion of parasitized hosts; therefore, gregarious parasitoids were considered a single parasitism event. First, we tested whether the parasitism rate differed among tree species. Therefore, we used a logistic regression model with binomial distribution and link function logit. Because the parasitism rate was calculated from different numbers of individuals, different sample sizes (i.e., number of hosts) were considered: weighted least squares method was used in the regression model to maximize the efficiency of parameter estimation (i.e., proportions calculated from a higher number of hosts had a greater effect on model parameters). The other model settings were the same as in the case of the previous GLMs. To investigate the effect of canopy level and season on parasitism rate, we used generalized linear mixed‐effect model (GLMM) with binomial distribution and link function logit in the package lme4 (Bates, Maechler, Bolker, & Walker, 2015). Based on the results of the logistic regression model, tree species was set as a random effect. To compensate for sampling effort, the relationship between mean parasitism and the linear predictor of the model was offset by a common logarithm of the leaf area. Therefore, parasitism was defined as parasitism rate per given leaf area. Moreover, as in the case of logistic regression model, different sample sizes (i.e., numbers of hosts) were considered. We used two explanatory variables (canopy level and season) and the interaction between canopy level and tree species. The partial effect of each explanatory variable was tested using likelihood ratio (LR) analysis of deviance with *F* statistics.

In addition, we estimated the vertical preferences of the most abundant parasitoid taxa (Tachinidae, Ichneumonidae, Braconidae, and Chalcidoidea) as the proportion of parasitized hosts to the number of all potential hosts for each parasitoid group, based on observed host–parasitoid links in our dataset. Differences in proportions were subsequently tested with a series of proportion tests (Newcombe, 1998), which were performed for each parasitoid group among canopy levels. For the analyses, we only used host species that were parasitized at least three times by a particular taxonomic group.

To test for relationships among host and parasitoid abundance and leaf area, we used Spearman's rank correlation method. Relationships between host and parasitoid abundance, and between host and parasitoid species richness, were investigated using Spearman's rank partial correlation with sample size (i.e., leaf area and total abundance, respectively) as a covariate (Hollander & Wolfe, 1973).

#### 2.3.4 Differences in species composition

To test whether species composition of hosts and parasitoids differed among canopy levels and tree species, we used analysis of similarities (ANOSIM) in the package vegan (Oksanen et al., 2016). The dissimilarity matrix was calculated from the species matrix using function *vegdist* based on Bray–Curtis distance (Bray & Curtis, 1957). The significance of the ANOSIM analysis was assessed by the permutation test (1,000 permutations). *R* values close to 1 indicate high separation between levels of selected factors (i.e., tree species and canopy level), whereas values close to 0 indicate low separation. The test statistic *R* is calculated as follows: *R* = (*r*_B_^ ^− *r*_W_)/(*M*/2), where *r*_B_ is the average of rank similarities among samples from different sites, *r*_W_ is the average of rank similarities among samples within sites, and *M* = *n*(*n *− 1)/2, where *n* means number of samples. We also investigated whether the variability (variance) inside the selected groups (canopy levels and tree species) differed from the other groups. Therefore, we calculated the average distance of group members to the group centroid using permutational analysis of variance (PERMANOVA) with 500 permutations to test whether the variances of one or more groups differed (Oksanen et al., 2016).

#### 2.3.5 Host feeding mode

To investigate the vertical preferences of exposed and semiconcealed hosts and their parasitoids, we tested differences in density of host guilds and their parasitism rate among canopy levels. Differences were tested using a series of proportion tests (Newcombe, 1998). To assess the differences in species composition between parasitoid communities (exposed vs. semiconcealed hosts), we calculated the Bray–Curtis dissimilarity index (Bray & Curtis, 1957) using function *vegdist* of package vegan (Oksanen et al., 2016).

#### 2.3.6 Parasitoid–host specificity

Host specificity of the most abundant parasitoid taxonomic groups (Tachinidae, Chalcidoidea, Braconidae, and Ichneumonidae) was calculated as the average host phylogenetic diversity (PD; Faith, 1992), using function *pd* in the package picante (Kembel et al., 2010). PD is the sum of all phylogenetic branch lengths connecting species in a community and was implemented as a measure of host specificity (Poulin, Krasnov, & Mouillot, 2011). For each parasitoid species, we first calculated the PD of its hosts as a sum of all branch lengths connecting the focal set of its host species in a phylogenetic tree (Supporting Information Table S2, Figure S1). Tree was generated from COI sequences of 176 leaf‐chewer species involved. Host species for which a DNA barcode sequence was not available (*Nematus umbratus*,* Apethymus cerris*) were substituted by a congener, while one species (*Eupareophora exarmata*) had to be excluded from the final tree due to unavailability of a congeneric sequence. Sequences were generated during our study or downloaded from BOLD (IDs of respective sequences are included in tip labels of the tree). Sequences were aligned in MAFFT version 7 (Katoh & Standley, 2013) on the MAFFT server (http://mafft.cbrc.jp/alignment/server/), and the tree was constructed using Randomized Axelerated Maximum Likelihood method (RAxML). The RAxML analyses were conducted on the CIPRES computer cluster using RAxML‐HPC BlackBox 7.6.3 (Stamatakis, 2006) with default settings. Tree was subsequently converted to ultrametric and visualized in FigTree (Rambaut, 2014). Because PD is positively correlated with species richness, we divided the PD index by the number of host species utilized. Subsequently, we calculated the mean PD value for all parasitoid species within a particular parasitoid group. Parasitoids exploiting three or more individuals of the same species and no other species were considered monophagous, and their PD values were set to zero, while single‐ and doubletons were excluded from the analysis (Symons & Beccaloni, 1999).

#### 2.3.7 Food web metrics

To assess the changes in leaf‐chewing host–parasitoid food web structure in a vertical canopy gradient, we calculated standard metrics that characterize the complexity and structure of the entire food web, and which reflect the degree of network specialization (Morris, Gripenberg, Lewis, & Roslin, 2014; Tylianakis, Tscharntke, & Lewis, 2007). We focused on quantitative metrics that reflect interaction network properties and that are more robust against variation in sampling intensity, matrix size, and symmetry than qualitative ones (van Veen et al., 2006). Specifically, we used: (a) number of compartments (subsets of the web not connected to other compartments); (b) weighted nestedness based on overlap and decreasing fill (nestedness quantifies whether a given sequence of columns [rows] shows decreasing marginal totals, that is, incidences or richness); (c) weighted quantitative linkage density (linkage density is the weighted diversity of interactions per species); (d) weighted quantitative connectance (connectance is the weighted realized proportion of possible links, calculated as quantitative linkage density divided by the number of species in the network); (e) weighted quantitative interaction evenness (interaction evenness is a measure of the uniformity of energy flows along different pathways); (f) weighted quantitative network specialization index $H_{2}^{\prime }$ (degree of specialization among hosts and parasitoids across an entire network); (g) weighted quantitative generality (generality is the mean effective number of hosts per parasitoid weighted by their marginal totals); (h) weighted quantitative vulnerability (vulnerability is the mean effective number of parasitoids per host species, weighted by their marginal totals); and (i) weighted quantitative modularity (modularity is the degree to which a quantitative network can be divided into modules, within which within‐module interactions are more prevalent than between‐module interactions). Full formulae and software details are provided in Almeida‐Neto and Ulrich (2011), Dormann, Fründ, Blüthgen, and Gruber (2009), and Dormann and Strauss (2014).

To calculate the metrics, we post hoc selected 14 trees from six species (*Q. cerris, Quercus robur, C. betulus, A. campestre, Fraxinus* spp., and *Ulmus laevis*) that met our criteria: (i) was sampled at least twice during one season and (ii) had at least two host and two parasitoid species per canopy level. Food web metrics were calculated for each canopy level (42 networks) and were fitted by a series of GLMs. Poisson distribution and link function log were used when the values of the food web metrics were integers, and inverse Gaussian distribution and link function log were used when the food web metrics were real numbers. As matrix size (i.e., the total number of interactions recorded between individuals) might affect many of the metrics (Morris et al., 2014; Rodriguez‐Girones & Santamaria, 2006), we controlled for matrix size (which varied from two to 219 interactions) in our models. First, we regressed the food web metrics on a common logarithm of matrix size. Then, we used residuals from the previous regressions as response variables, and we used tree species, tree height, canopy level, and their interactions as explanatory variables. The partial effect of each explanatory variable was tested using LR analysis of deviance with the chi‐square statistics. Food web metrics were calculated in the package bipartite (Dormann et al., 2009), using the *empty.web* = *false* option to account for hosts that were present but not parasitized (Morris et al., 2014).

## 3 RESULTS

### 3.1 The dataset

We sampled 2,494 m^2^ of foliage from 59 trees of eight species. We collected 10,225 (*n*) leaf‐chewing larvae representing 177 species (*S*; these fed on host plant they were sampled from), and we recorded 1,256 parasitism events (including 28 hyperparasitoids), resulting in 2,286 reared or dissected parasitoids representing 143 putative species (Table 1).

**Table 1:** Insects sampled from a 0.2 ha broadleaf deciduous forest plot in the Czech Republic between 2013 and 2015 (*n* = number of individuals, *S* = number of species)

Tree species	Tree no	Leaf area (m^2^)	Avg. height (m)	Leaf‐chewers		Parasitoids^a^	
				*n*	*S*	*n*	*S*
*Acer campestre*	16	320.6	18.8	1,977	83	237	53
*Carpinus betulus*	13	735.5	18.3	904	83	165	50
*Fraxinus* spp.^b^	9	568.4	32.7	669	42	57	25
*Quercus cerris*	7	246.1	39.5	2,397	94	493	63
*Quercus robur*	5	450.4	27.2	3,951	84	244	49
*Ulmus laevis*	6	101.0	13.4	217	34	34	15
*Tilia cordata*	3	71.9	17.1	110	23	26	15
Total	59	2,493.9		10,225	177	1,256	143

Notes

a Parasitoid rearing events, hyperparasitoids included (28 individuals of one species from Perilampidae).

b *Fraxinus* spp. were represented by eight individuals of *F. angustifolia* and one individual of *F. excelsior*.

The community of 10,225 leaf‐chewers was represented by 24 families, which were dominated by Geometridae (53.3%, *n* = 5,450, *S* = 35), Noctuidae (15.46%, *n* = 1,581, *S* = 27), Tortricidae (6.54%, *n* = 669, *S* = 30), Bucculatricidae (6.07%, *n* = 621, *S* = 2), Tenthredinidae (5.43%, *n* = 555, *S* = 14), and Lymantriidae (3.71%, *n* = 379, *S* = 6). The remaining 18 families were represented by 8.23% of the individuals. We were not able to identify 128 (1.25%) and 513 (5.02%) individuals to the family and species level, respectively.

The community of 1,256 parasitoids was represented by seven families and was dominated by Tachinidae (41.0%, *n* = 515, *S* = 21), Braconidae (22.45%, *n* = 282, *S* = 46), Ichneumonidae (21.82%, *n* = 274, *S* = 50), and Eulophidae (6.21%, *n* = 78, *S* = 19). The remaining three families were represented by 2.63% of individuals. We were not able to identify 74 (5.89%) and 144 (11.46%) individuals to the family and species level, respectively. For further details about the host and parasitoid species, see Supporting Information Tables S2 and S3.

### 3.2 Insect density

**Figure 1:**
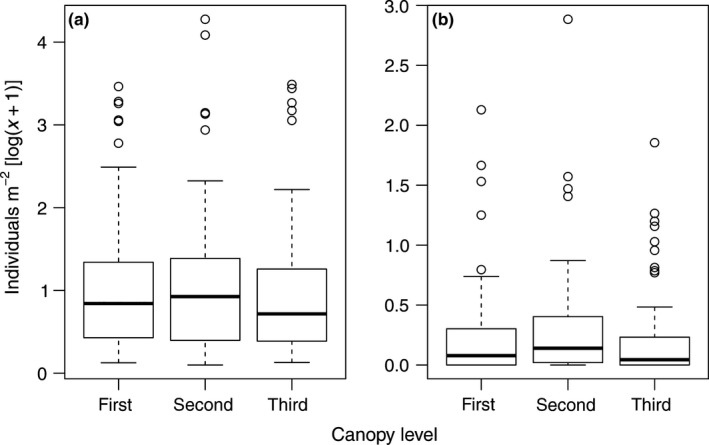
Box plots showing the density of (a) hosts and (b) parasitoids in each canopy level

**Figure 2:**
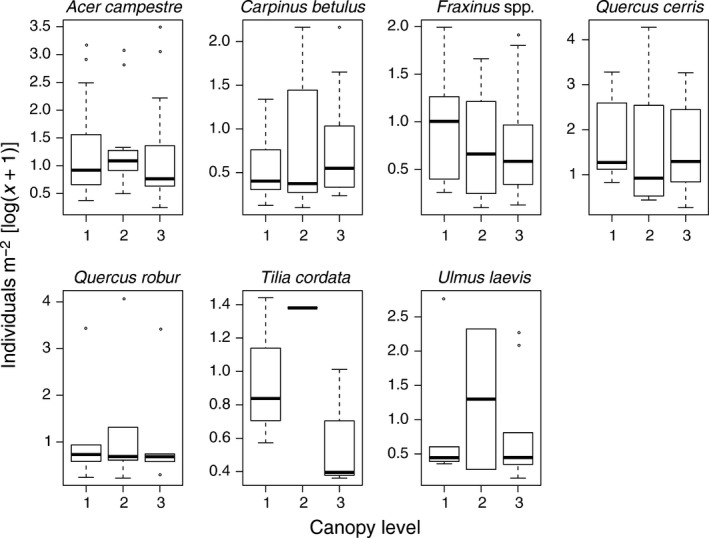
Box plots showing the density of hosts in each canopy level (1 = first, 2 = second, and 3 = third) and tree species

The highest density of both hosts and parasitoids was found in the second level, followed by the first and third canopy levels (Figure 1). However, a significant difference was only found for hosts (*F*_2,134_ = 3.06, *p *=* *0.04), not for parasitoids (*F*_2,134_ = 1.79, *p *=* *0.17). Densities differed significantly among tree species (hosts: *F*_5,147_ = 5.73, *p *<* *0.001; parasitoids: *F*_5,147_ = 6.68, *p *<* *0.001). Interaction between canopy level and tree species was significant for hosts (*F*_18,134_ = 2.66, *p *<* *0.001) (Figure 2), but not for parasitoids (*F*_18,134_ = 1.19, *p *=* *0.27). Densities of both groups decreased as the season progressed (hosts: *F*_1,162_ = 9.54, *p *<* *0.001; parasitoids: *F*_1,162_ = 82.68, *p *<* *0.001). The highest mean host densities were recorded on *Q. cerris*, followed by *Q. robur*,* A. campestre*, and *U. laevis* (10.60, 8.75, 4.80, and 2.89 hosts/m^2^, respectively). In comparison, the lowest mean host densities were found on *C. betulus*, followed by *T. cordata* and *Fraxinus* spp. (1.53, 1.57, and 1.78 hosts/m^2^, respectively). The highest densities of parasitoids were recorded on *Q. cerris*, followed by *A. campestre*,* Q. robur*, and *U. laevis* (2.18, 0.58, 0.54, and 0.45 parasitoids/m^2^, respectively). In comparison, the lowest densities of parasitoids were recorded on *Fraxinus* spp. followed by *C. betulus* and *T. cordata* (0.15, 0.28, and 0.37 parasitoids/m^2^, respectively). Host abundance was positively correlated with leaf area (*ρ* = 0.424, *S* = 423 620, *df* = 162, *p *<* *0.001), whereas parasitoid abundance was positively correlated with both leaf area (*ρ* = 0.252, *S* = 550 060, *df* = 162, *p *=* *0.001) and host abundance (*ρ* = 0.706, *t* = 12.66, *df* = 161, *p *<* *0.001).

### 3.3 Insect diversity

**Figure 3:**
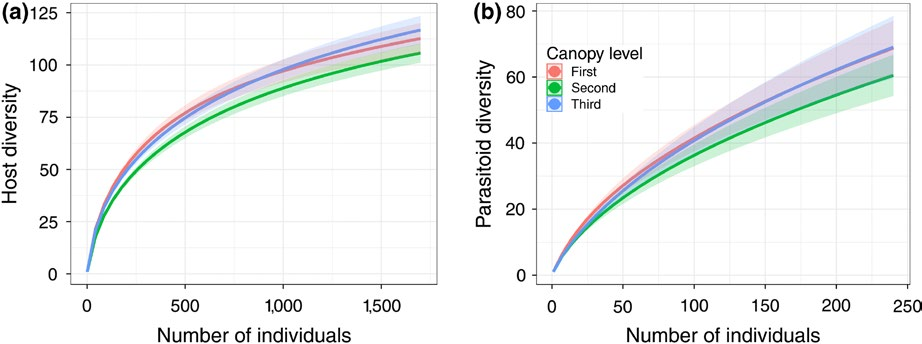
Individual‐based rarefaction curves of species diversity of (a) hosts and (b) parasitoids in each canopy level pooled across all tree species. Solid lines represent rarefaction; shaded areas represent 95% confidence intervals

**Figure 4:**
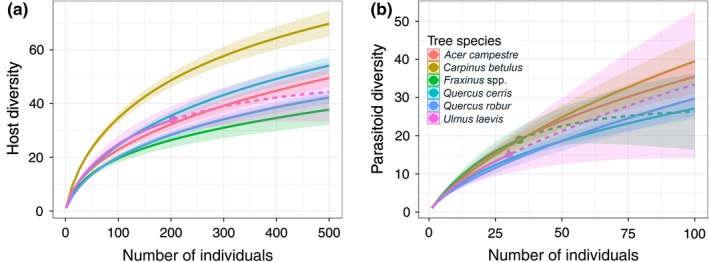
Individual‐based rarefaction curves of species diversity of (a) hosts and (b) parasitoids among tree species. Solid lines represent rarefaction, dashed lines represent extrapolations, color points represent sampling extent, and shaded areas represent 95% confidence intervals

We found significant differences in the diversity among canopy levels for hosts (*F*_2,133_ = 3.48, *p *=* *0.03), but not for parasitoids (*F*_2,133_ = 1.65, *p *=* *0.196). Rarefaction revealed the highest diversity of both hosts and parasitoids in the first and third canopy levels, while the second level harbored the lowest diversity (Figure 3). However, this pattern was only noticeable in the tallest tree species (*Q. cerris*,* Q. robur*, and *Fraxinus* spp.) (Supporting Information Figure S2). Species diversity was significantly different among tree species (host: *F*_6,153_ = 8.56, *p *<* *0.001; parasitoids: *F*_6,153_ = 2.23, *p *=* *0.042). We also detected a significant interaction between canopy levels and tree species for hosts (*F*_18,133_ = 5.32, *p *<* *0.001), but not for parasitoids (*F*_18,133_ = 0.96, *p *=* *0.512). Based on the rarefaction curves, the most species‐rich host assemblages were found on *C. betulus*, followed by *Q. cerris*,* A. campestre*, and *U. laevis*. In comparison, the least species‐rich assemblages were found on *Fraxinus* spp. and *Q. robur* (Figure 4a). Tree species‐related differences were less distinct for parasitoids (Figure 4b). The species richness of both groups decreased as the season progressed (hosts: *F*_3,151_ = 57.41, *p *<* *0.001; parasitoids: *F*_3,151_ = 49.51, *p *<* *0.001). Parasitoid species richness was positively correlated with host species richness (*ρ* = 0.158, *t* = 2.03, *df* = 161, *p *=* *0.044).

### 3.4 Parasitism rate

**Figure 5:**
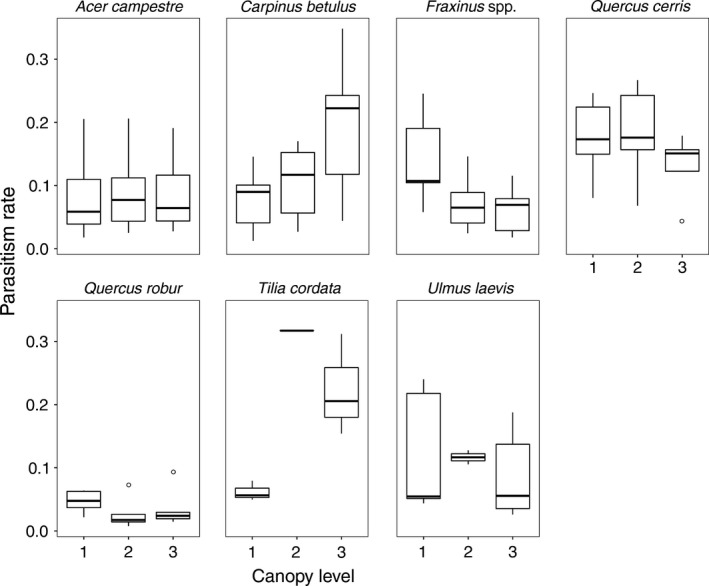
Differences in parasitism rates (proportion of parasitized hosts) in each canopy level (1 = first, 2 = second, and 3 = third) for individual tree species. Predicted values of the binomial generalized linear mixed‐effect model are shown on the *y*‐axis

Overall, the parasitism rate significantly differed among canopy levels (LRT = 9.293, *p *<* *0.01) and was highest in the first canopy level (14.99%), followed by the second and third levels (12.19% and 10.91%, respectively). The parasitism rate differed among tree species (LRT = 22.63, *p *<* *0.001), with the highest rates on *T. cordata*, followed by *Q. cerris*,* C. betulus*, and *U. laevis* (23.6%, 20.6%, 18.3%, and 15.6%, respectively). The lowest rates were documented on *Q. robur*, followed by *Fraxinus* spp. and *A. campestre* (6.2%, 8.5%, and 12%, respectively). The parasitism rate was also significantly different with respect to canopy level and tree species (LRT = 57.71, *p *<* *0.001) (Figure 5). The parasitism rate decreased as the season progressed (LRT = 5.93, *p *=* *0.01), every month from May to August (13.92%, 10.96%, 5.41%, 4.28%, respectively).

**Figure 6:**
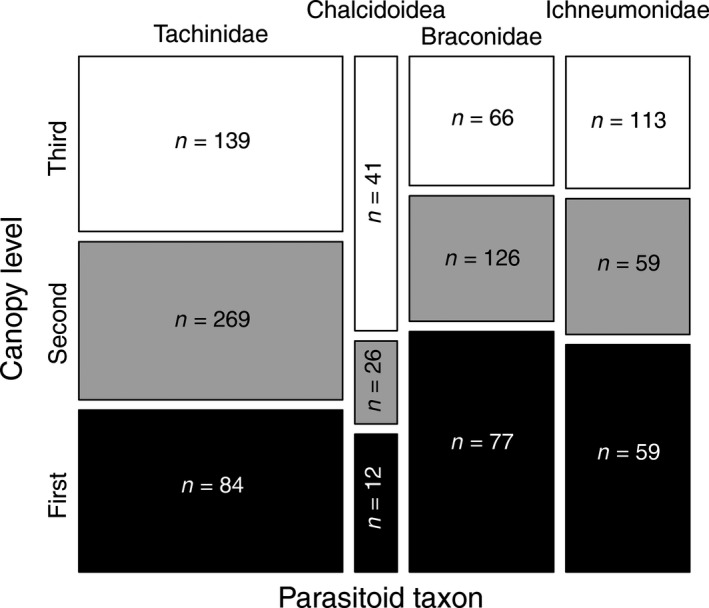
Most abundant parasitoid taxa canopy‐level preferences expressed as a parasitism rate calculated as a proportion of parasitized hosts to the number of all potential hosts for each parasitoid group, based on observed host–parasitoid species links. Column height represents parasitism rate, while column width represents the abundance of parasitoids within each taxonomic group. Total abundances of parasitized hosts among canopy levels are shown (*n*)

Regarding parasitoid canopy‐level preferences, we found a significant vertical stratification pattern in parasitism rate for Chalcidoidea (*χ* ^2^ = 26.25, *df* = 2, *p *<* *0.001), which preferred third‐level hosts (55%), and for Braconidae and Ichneumonidae (*χ* ^2^ = 24.46, *df* = 2, *p *<* *0.001 and *χ* ^2^ = 12.84, *df* = 2, *p *=* *0.002; respectively), which preferred first‐level hosts (49% and 46%, respectively). Only Tachinidae exploited their hosts evenly, with no significant canopy‐level preference (*χ* ^2^ = 1.064, *df* = 2, *p *=* *0.587; Figure 6).

### 3.5 Differences in species composition

Host species composition was more distinct among, rather than within, tree species (ANOSIM; R = 0.204, Supporting Information Table S4, Figure S3). However, in parasitoids, no such relationship was detected (R = 0.062). Regarding the vertical gradient, host and parasitoid assemblages were similar (*R* = −0.017 and *R* = −0.006, respectively) among, and within, canopy levels (Supporting Information Figure S3).

### 3.6 Host feeding mode

We recorded 8,375 exposed (104 species from 12 families) and 1,098 semiconcealed (70 species from 10 families, predominantly Tortricidae and Psychidae) leaf‐chewers (Supporting Information Table S3). Parasitoids of exposed and semiconcealed feeders were represented by 75 and 41 species, respectively (Bray–Curtis index, 80.1% dissimilarity), while 19 species exploited both guilds (Supporting Information Table S2).

We found significant differences in the densities of both exposed (*χ* ^2^ = 490.8, *df* = 2, *p *<* *0.001) and semiconcealed feeders (*χ* ^2^ = 83.72, *df* = 2, *p *<* *0.001) among canopy levels. The highest density was recorded in the second level for both exposed and semiconcealed feeders (4.88 and 0.55 individuals/m^2^, respectively), while the lowest was recorded in the first level (2.18 and 0.22 individuals/m^2^, respectively). However, the density of exposed feeders decreased significantly between the second and third level (*χ* ^2^ = 198.6, *df* = 1, *p *<* *0.001), from 4.88 to 2.94 individuals/m^2^. In comparison, for semiconcealed feeders, this decline (from 0.55 to 0.52 individuals/m^2^) was not significant (*χ* ^2^ = 0.2413, *df* = 1, *p *=* *0.623). The overall parasitism rate between exposed (12.85%) and semiconcealed feeders (13.02%) did not differ significantly (*χ* ^2^ = 0.0134, *df* = 1, *p *=* *0.908); however, the parasitism rate of exposed feeders varied significantly among canopy levels (*χ* ^2^ = 19.73, *df* = 2, *p *<* *0.001), with the highest rate occurring in the first level (16.3%). These differences were not significant (*χ* ^2^ = 5.122, *df* = 2, *p *=* *0.077) in semiconcealed feeders. For semiconcealed feeders, we recorded a clear decline in parasitism rate (15.1%–10.3%) between the second and third levels (*χ* ^2^ = 4.448, *df* = 1, *p *=* *0.035), while the decline recorded for exposed feeders (12.3%–11.8%) was not significant (*χ* ^2^ = 0.398, *df* = 1, *p *=* *0.528).

### 3.7 Parasitoid–host specificity

Individual parasitoid groups exhibited different average host PD, with the lowest values being obtained for Chalcidoidea (PD = 26.3) and Tachinidae (PD = 34.5), suggesting higher host specificity. Higher values were obtained for Braconidae (PD = 47.0) and Ichneumonidae (PD = 72.4), suggesting lower host specificity.

### 3.8 Food web structure

Our food webs document 57 host and 106 parasitoid species involved in 744 trophic interactions. From all network metrics tested, only linkage density and vulnerability significantly differed among canopy levels (*df* = 2, Δ*G* = 7.62, *p *=* *0.022; *df* = 2, Δ*G* = 9.60, *p *<* *0.01, respectively). Connectance and the number of compartments significantly differed among trees of different heights (*df* = 1, Δ*G* = 4.02, *p *=* *0.044; *df* = 1, Δ*G* = 8.15, *p *<* *0.01, respectively). Moreover, there were significant differences in the linkage density, $H_{2}^{\prime }$, generality, connectance, modularity, and number of compartments among individual tree species (*df* = 5, Δ*G* = 12.43, *p *=* *0.029; *df* = 5, Δ*G* = 4.66, *p *=* *0.011; *df* = 5, Δ*G* = 12.84, *p *=* *0.024; *df* = 5, Δ*G* = 21.19, *p *<* *0.001; *df* = 4, Δ*G* = 9.82, *p *=* *0.043; *df* = 5, Δ*G* = 15.11, *p *<* *0.01, respectively). In comparison, a significant interaction between tree species and tree height was found for interaction evenness, $H_{2}^{\prime }$, generality, and nestedness. For interaction evenness and nestedness, there was a significant interaction between canopy level and tree height and between canopy level and tree species (Supporting Information Tables S5 and S6 and Figures S4 and S5).

## 4 DISCUSSION

### 4.1 Density and diversity

In general, the abundance of hosts was positively correlated with leaf area. As expected, there were significant differences in host densities among individual tree species. However, contrary to our expectations, the highest host density was recorded in the second canopy level, followed by the first and third levels. The abundance of temperate forest caterpillars varies with host tree species (Murakami et al., 2007); however, studies on the stratification of caterpillars within a vertical canopy gradient yielded inconsistent results (reviewed by Ulyshen, 2011). One of the important determinants of how insects are distributed locally in temperate and tropical forests is predation (e.g., Olson, 1992; Šipoš, Drozdová, & Drozd, 2013). The predation pressure on caterpillars along a vertical gradient in a temperate floodplain forest is greatest in the understory and the upper canopy (Šipoš, Suchánková, & Drozd, unpublished data). Therefore, in our study, the observed stratification of host density might stem from the stratification of resources and microclimatic conditions, as suggested by our original hypothesis, but also from higher predation rates in the first and third canopy levels. Moreover, we found a significant interaction between tree species and canopy level as factors affecting host density distribution. Interestingly, hosts on smaller tree species (*A. campestre*,* C. betulus*,* T. cordata*) were generally the least abundant in the first level, with the opposite trend being detected on taller tree species (*Quercus* spp., *Fraxinus* spp.). This pattern might be explained by the uneven distribution of predation pressure. The first level of smaller tree species is close to understory with high predation rates, whereas the canopy of taller trees is much higher and is not in direct contact with the understory. Therefore, predation might be less frequent in the first level of taller species, resulting in higher host densities in the first level.

Herbivore species composition was determined by tree species, as expected, but not by canopy level. However, the vertical gradient was manifested by host diversity, which was highest in the first and third canopy levels; however, this trend was only marked in the tallest tree species. Variation in microclimatic conditions and resource quality might impede the diversity of Lepidoptera along a vertical gradient (Schulze, Linsenmair, & Fiedler, 2001). Due to its higher foliage quality, the understory of temperate forests harbors more species‐rich lepidopteran assemblages than the canopy (Hirao et al., 2009; Le Corff & Marquis, 1999). Therefore, the higher diversity recorded in the first level might be due to the involvement of understory species, supporting our original hypothesis. The higher diversity of the third level might be explained by the presence of species‐rich semiconcealed feeders, which were considerably less numerous in the first level, supporting the original hypothesis of the competitive advantage of the concealed feeders in the harsh upper canopy environment (Chaij et al., 2016; Paniagua et al., 2009; Ribeiro & Basset, 2007). However, in our study, due to the absence of the upper canopy per se, differences in the ecological conditions (e.g., solar radiation, temperature, wind speed; Basset, Horlyck, et al., 2003; Parker, 1995) between the second and third levels were blurred. For example, in the third level, the harsher conditions of the upper canopy of taller trees were merged with more moderate conditions in smaller trees because their third level was shaded. Consequently, differences in density and diversity toward the upper canopy might be more marked.

The abundance and richness of parasitoids were positively correlated with the abundance and richness of hosts, suggesting that high host diversity promotes high parasitoid diversity in a leaf‐chewer–parasitoid assemblage via a varied niche base. A similar pattern was found by Paniagua et al. (2009) for gall‐forming insects and by Tylianakis, Tscharntke, and Klein (2006) for cavity‐nesting Hymenoptera. Vertical patterns in parasitoid density and diversity followed those found in hosts, but were not significant, probably because of the generally lower numbers of parasitoids compared to hosts. Similarly, tree species‐related differences in parasitoid diversity and species composition were not significant. Based on the analysis of host specificity of the four most abundant parasitoid groups, Chalcidoidea were the most host‐specific, followed by Tachinidae, Braconidae, and Ichneumonidae. In accordance with our original prediction, the high host specificity of Tachinidae was reflected in the vertical use of available hosts, as they closely followed the distribution of their hosts, with no apparent preference for canopy level. Thus, there appeared to be no environmental constraints within the canopy for this group. Moreover, for species with high dispersal abilities, patterns of vertical distribution are mainly determined by the availability of resources (Ulyshen, 2011), and tachinid flies are known as highly mobile fliers (e.g., Stireman et al., 2012). However, this pattern was not observed in the highly host‐specific Chalcidoidea, as they exhibited a clear preference for the third canopy level. Accordingly, the most abundant family of chalcid wasps in our dataset, Eulophidae, is in tropical forests actually known as high fliers, being active above the upper canopy (Compton et al., 2000). In comparison, the less host‐specific Ichneumonidae and Braconidae used their hosts nonrandomly, preferring the first canopy level. This phenomenon suggests some environmental (Basset, Hammond, et al., 2003; Ulyshen, 2011) and/or life history constraints within these groups, such as low dispersal capacity (Godfray, 1994) and/or limited number of eggs carried by females combined with the proximity of overwintering sites to the first canopy level (Chaij et al., 2016; Heimpel, Mangel, & Rosenheim, 1998). Moreover, most parasitoids depend on sugar resources, due to their high activity (Shaw, 2006), with sugar feeding increasing host encounter rates (Forsse, Smith, & Bourchier, 1992). In contrast to that documented for the tropics, the availability of sugar resources in temperate forests varies with height, with floral nectar being more readily available in the understory (Ulyshen, 2011). This phenomenon might also explain why less host‐specific parasitoid taxa preferred the first canopy level.

### 4.2 Parasitism rate

Comparisons of vertical patterns of parasitism are limited, as most studies only compared the canopy with the understory. Moreover, no previous study focused on leaf‐chewing assemblages. Inconsistent results obtained by previous studies suggest that the vertical patterns of parasitism are guild‐, latitude‐, or site‐dependent (Chaij et al., 2016; Morris et al., 2015; Paniagua et al., 2009; Sobek, Tscharntke, Scherber, Schiele, & Steffan‐Dewenter, 2009). The parasitism rate decreased as the season progressed, in accordance with another study on leaf‐chewing Lepidoptera in a temperate forest (Le Corff, Marquis, & Whitfield, 2000). As we expected, the overall parasitism rate generally decreased from the first to the third level and significantly differed among tree species. Individual tree species shared many host species, as suggested by the Bray–Curtis dissimilarity index, with individual herbivore species’ vulnerability to parasitoid attack varying in relation to host plants. Thus, host plants influence parasitism rates for herbivore assemblages and individual species through various mechanisms, such as specific plant volatiles and different apparency of hosts to predators (see Lill et al., 2002). However, as in the case of density, an interesting trend occurred in the vertical patterns of parasitism when viewing tree species according to their average heights. For smaller tree species, parasitism increased from the first toward the third level, whereas taller trees species showed the opposite trend. The abiotic conditions of the upper canopy are assumed to limit parasitoid activity in the uppermost layers (Fernandes & Price, 1992); therefore, the presence of the upper canopy probably promoted lower parasitism rates in the third level of taller trees. Moreover, predation negatively affects host density, with parasitoids also being eaten by predators along with their hosts. In such case, parasitoids act as prey in intraguild predation (Rosenheim, Kaya, Ehler, Marois, & Jaffee, 1995). However, prey might temporarily or spatially avoid areas where predators are abundant or particularly active (Olson, 1992). Moreover, parasitoids might aggregate in patches with more hosts, which increases their efficiency, resulting in higher parasitism rates (Murdoch & Stewart‐Oaten, 1989). Therefore, we suggest that the parasitism rate was stratified by the parasitoid spatial avoidance of the level close to the understory and of the upper canopy. The “middle canopy” (i.e., second and third level of smaller trees, and first and second level of higher trees) might, thus, provide microhabitat refuge for both herbivores and their parasitoids, resulting in higher parasitism rates.

As expected, the species composition of the parasitoid community largely differed between exposed and semiconcealed hosts. However, surprisingly, the parasitism rate did not significantly differ between the two guilds, contrasting with that found in the tropics, in which the latter group suffers higher parasitism (Gentry & Dyer, 2002; Hrcek et al., 2013). Moreover, an interesting trend occurred when comparing vertical trends in parasitism between guilds. Contrary to our expectations, both groups were most abundant in the second level, but only exposed feeders significantly decreased in density toward the third level. Nevertheless, for exposed feeders, we recorded only a slight decrease in parasitism toward the third level, whereas semiconcealed feeders experienced a markedly lowered parasitism rate in the third level, compared to the second. These results suggest that, even though semiconcealed feeders are less protected than concealed ones, they still have an advantage over exposed feeders, at least, in temperate forests. In brief, because they are less vulnerable to harsh conditions and predators (Novotny et al., 2006; Ribeiro & Basset, 2007), they can occupy higher forest levels, reducing parasitism.

### 4.3 Quantitative food webs

Despite our predictions, the food web structure within the vertical gradient was generally consistent; network metrics did not significantly differ among canopy levels, with the exception of linkage density and vulnerability. The only other study of herbivore–parasitoid food webs in the vertical canopy gradient of the temperate forest found that food web complexity decreased with canopy height in a monotypic beech forest (Chaij et al., 2016). However, as we expected, there were significant differences in food web structure among individual tree species, as reflected by linkage density, $H_{2}^{\prime }$, generality, connectance, modularity, and the number of compartments. This result suggests that host tree species are more important than the vertical gradient for shaping the food web structure. Nevertheless, some subtle vertical patterns occurred. For instance, connectance decreased steadily from the lowest to the highest trees, regardless of tree species, which might be due to changes in the prevalence of specialists or generalists (Dunne, 2006). Moreover, generality was highest and $H_{2}^{\prime }$ was lowest in the tallest tree species, suggesting lower specialization in the upper canopy (Morris et al., 2014). High generality, recorded in the tallest tree species (*Fraxinus* spp. and *Q. cerris*), could be explained by the presence of parasitoids interacting with more host species within a particular web. For example, in *Fraxinus* spp.*,* a relatively high mean generality index was probably influenced by a single parasitoid species (BOLD:AAF6259, possibly *Lypha* sp.) exploiting four of 11 hosts in the interaction matrix. Similarly, we found one tachinid (BOLD:ACO3995, *Phorocera* sp.) and one braconid parasitoid (BOLD:ACU2814, *Protapanteles* sp.) on *Q. cerris* that exploited seven and four of 28 hosts, respectively. Although such parasitoids were also found on other tree species, they exploited a considerably lower proportion of host species; thus, their effect on the overall values of the generality index was much lower. Paradoxically, in our dataset, tachinids were mostly responsible for the lower specialization of networks in which they were involved, as one species usually interacted with more host species. However, they attacked closely related hosts, thus, exhibiting high level of host (phylo)specificity. This bias points out the need to interpret food web outputs with care, taking into account knowledge of the species involved and their phylogenetic relationships.

Because host species assemblages were largely similar among tree species, the mechanisms underlying tree species‐related differences in food web structure might be similar to those detected for parasitism rates (see Lill et al., 2002) and/or might stem from differences in the prevalence of specialists versus generalists (Leppänen, Altenhofer, Liston, & Nyman, 2012). Spatial dynamics in natural food webs probably operate alongside other coexistence mechanisms, such as competitive life history trade‐offs, forms of resource partitioning within local habitats and predation. In other words, ecological determinants of local species composition (Pillai, Gonzalez, & Loreau, 2011) or evolutionary constraints shaping community phylogeny (Leppänen et al., 2012) reflect the structure of the network. Thus, both the abundance and identity of the species involved reflect the food web structure of individual tree species.

Linkage density and vulnerability were the only metrics that differed among canopy levels, suggesting richer and more interactive food webs in the second canopy level. These patterns might result from the fact that the second level was dominated by the superabundant herbivore generalist *Operophtera brumata*, species, which was involved in most of the interactions, providing a wide niche base for various parasitoid species. Two other indices reflecting network structure (nestedness and modularity) did not show any clear trend. In trophic networks, decreased nestedness and increased modularity indicate a web structure where many interactions involve specialists, with each interacting with a small subgroup (Thébault & Fontaine, 2010). Because of the presence of semiconcealed feeders and their specific parasitoid assemblages in the second and third canopy levels, we expected higher modularity in these levels. However, modularity did not show any consistent vertical pattern, whereas vertical patterns of nestedness depended significantly on tree species and height. Thus, again, the specific conditions of individual tree species might be more important determinants of food web structure than the vertical canopy gradient.

## 5 CONCLUSIONS

Our results support those of Sobek et al. (2009). In brief, in temperate forests, tree diversity might be an important predictor of herbivore and parasitoid distribution patterns, and, hence, parasitism rates. However, our study generally contrasted with the prevailing paradigm of weak arthropod stratification in temperate forest canopies. We found significant stratification of host density, diversity, and parasitism rates, probably due to variation in biotic and abiotic conditions. In parasitoids, trends in density and diversity followed those of their hosts, but were not significant. Moreover, depending on the average height, each tree species created its own vertical gradient, shaping the stratification of insect density and parasitism rates. Consequently, the “middle zone” (i.e., the forest layer between the canopy level close to the understory and uppermost layer) was specific, with high insect densities and parasitism rates. However, the vertical gradient seemed to be less of a determinant for network structure, with the ecological characteristics of individual tree species probably being more important. However, our data from a single sampling plot cannot definitively generalize these patterns. Therefore, more replicates are needed to understand whether such patterns are widespread across sites and whether they are consistent for different herbivore guilds.

## CONFLICT OF INTEREST

None declared.

## AUTHOR CONTRIBUTIONS

PD and MŠ conceived the concepts and designed the methodology. MŠ, PP, NK, JŠ, and PD collected the data, MŠ, PP, and NK identified the insects. JŠ, MŠ, and PD analyzed the data. MŠ, HŠ, and JŠ wrote the manuscript. All authors gave final approval for publication.

## DATA ACCESSIBILITY

The insect data supporting the results are archived in Dryad Digital Repository (https://doi.org/10.5061/dryad.hk4948n). Specimen records with sequences from the whole host–parasitoid dataset are accessible on BOLD (https://doi.org/10.5883/DS-LANZHOT).
