# Supplementary material for: Vertical canopy gradient shaping the stratification of leaf‐chewer–parasitoid interactions in a temperate forest
Source: Ecol Evol. 2018 Jun 27;8(15):7297–311. doi: 10.1002/ece3.4194 (PMC6106176; doi:10.1002/ece3.4194)
Supplement: Supplementary file 1 [file ECE3-8-7297-s001.pdf]

Figure S1. Phylogenetic tree of host species used for calculations of parasitoid host specificity. Tree was generated from COI sequences of 176 leaf-chewer species involved. Host species for which a DNA barcode sequence was not available (*Nematus umbratus*, *Apethymus cerris*) were substituted by a congener, while one species (*Eupareophora exarmata*) had to be excluded from the final tree due to unavailability of a congeneric sequence. Sequences were generated during our study or downloaded from BOLD (IDs of respective sequences are included in tip labels of the tree). Sequences were aligned in MAFFT version 7 (Katoh & Standley, 2013) on the MAFFT server (http://mafft.cbrc.jp/alignment/server/), and a the tree was constructed using Randomized Axelerated Maximum Likelihood method (RAxML). The RAxML analyses were conducted on the CIPRES computer cluster using RAxML-HPC BlackBox 7.6.3 (Stamatakis, 2006) with default settings. Tree was subsequently converted to ultrametric and visualized in FigTree (Rambaut, 2014).

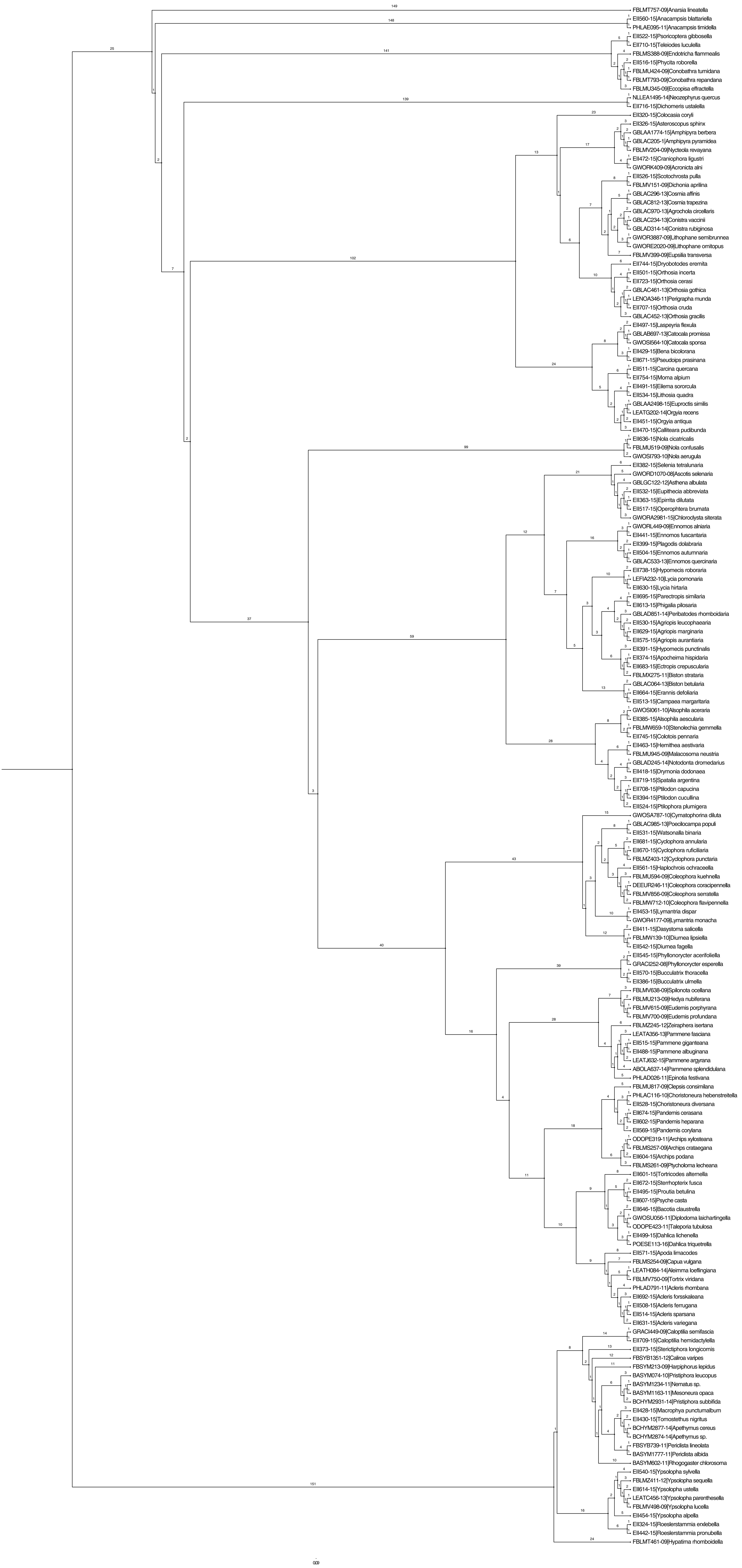

References

Katoh, K. & Standley, D.M. (2013) MAFFT Multiple Sequence Alignment Software Version 7: Improvements in Performance and Usability. *Molecular Biology and Evolution*, **30**, 772–780.

Rambaut, A. (2014) FigTree v1.4.2. Tree figure drawing tool. URL http://tree.bio.ed.ac.uk/software/figtree/.

Stamatakis, A. (2006) RAxML-VI-HPC: maximum likelihood-based phylogenetic analyses with thousands of taxa and mixed models. *Bioinformatics*, **22**, 2688–2690.
