# Supplementary material for: Vertical canopy gradient shaping the stratification of leaf‐chewer–parasitoid interactions in a temperate forest
Source: Ecol Evol. 2018 Jun 27;8(15):7297–311. doi: 10.1002/ece3.4194 (PMC6106176; doi:10.1002/ece3.4194)

Figure S2. Individual-based rarefaction curves of species diversity of hosts and parasitoids among canopy levels for individual tree species. Species diversity was estimated for rarefied and extrapolated samples with respect to sample size (number of individuals). Solid lines represent rarefaction, dashed lines represent extrapolation, color points represent sampling extent, and shaded areas represent 95% confidence intervals.

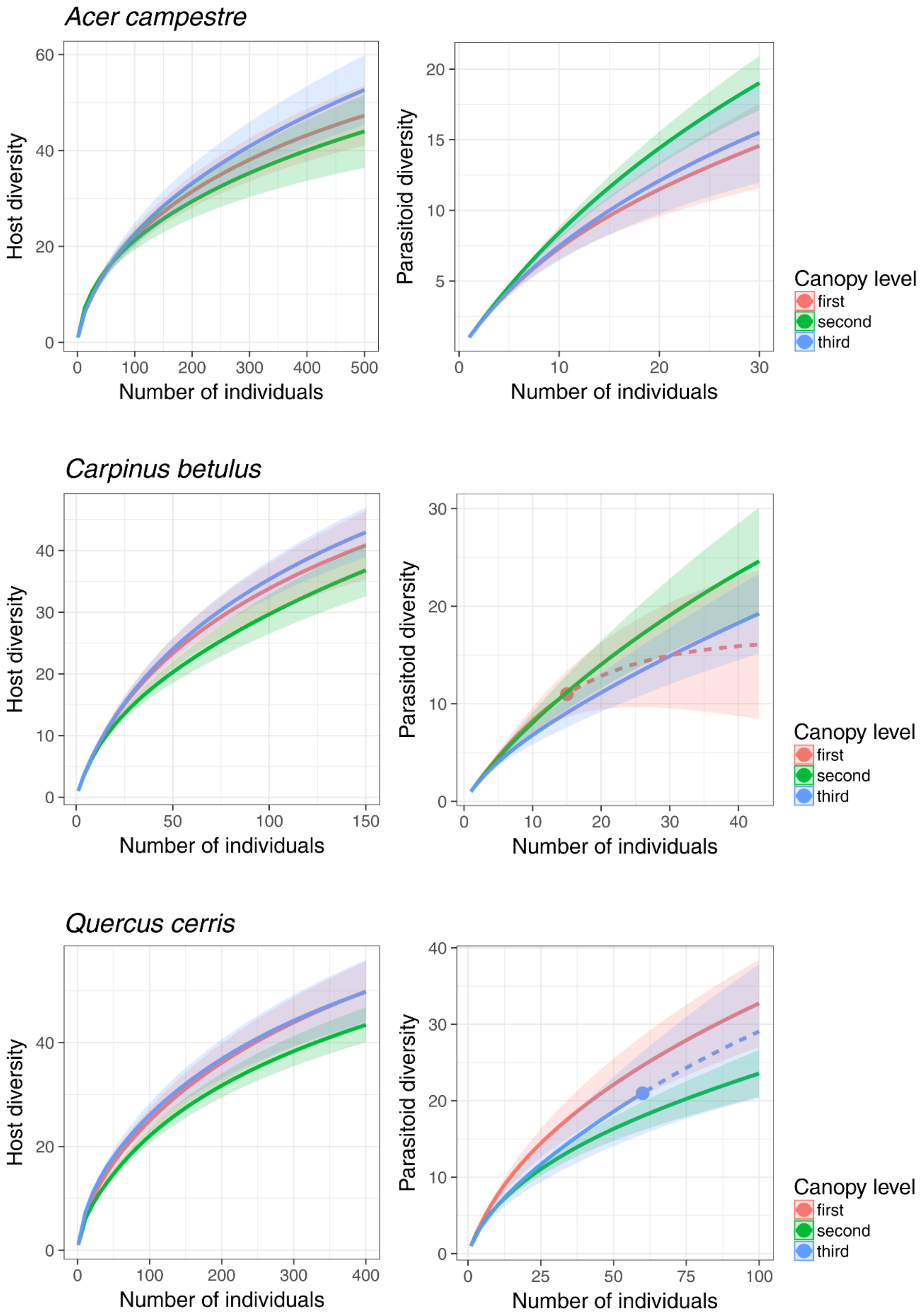

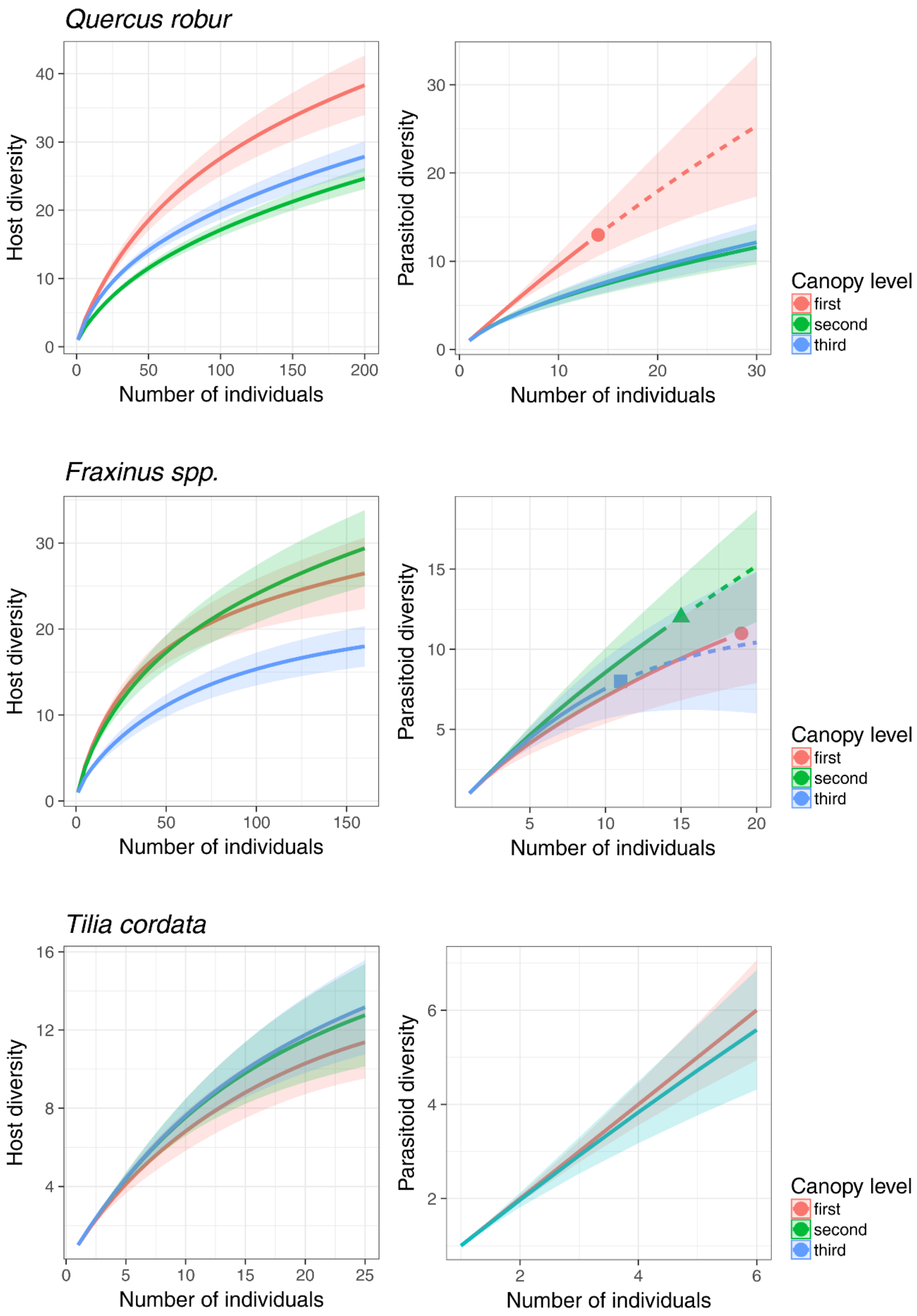

Quercus robur
Host diversity
Number of individuals
Parasitoid diversity
Number of individuals
Canopy level
first
second
third
Fraxinus spp.
Host diversity
Number of individuals
Parasitoid diversity
Number of individuals
Canopy level
first
second
third
Tilia cordata
Host diversity
Number of individuals
Parasitoid diversity
Number of individuals
Canopy level
first
second
third

Ulmus laevis

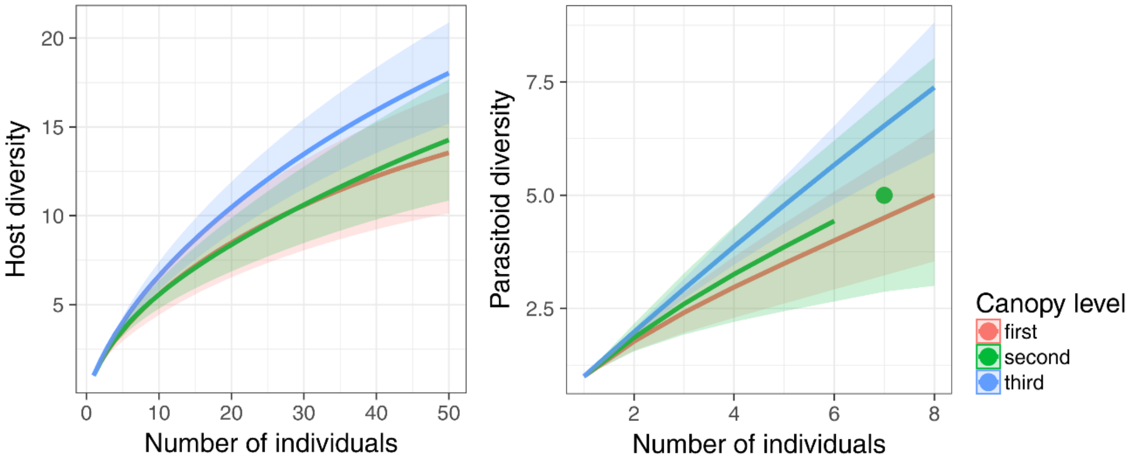

Supplement: Supplementary file 2 [file ECE3-8-7297-s002.pdf]
