# Supplementary material for: Vertical canopy gradient shaping the stratification of leaf‐chewer–parasitoid interactions in a temperate forest
Source: Ecol Evol. 2018 Jun 27;8(15):7297–311. doi: 10.1002/ece3.4194 (PMC6106176; doi:10.1002/ece3.4194)
Supplement: Supplementary file 3 [file ECE3-8-7297-s003.pdf]

Figure S3. Boxplots showing compositional dissimilarity in samples of (a) hosts, and (b) parasitoids. First boxplot in each figure shows the Bray-Curtis dissimilarity within entire dataset; other boxplots represent the same value in samples taken on particular tree species or canopy level. Median values, interquantile ranges, total ranges and outliers are shown. R-value measures difference among samples (zero means no difference among sets of samples), P-value measures statistical significance of R-value.

(a)

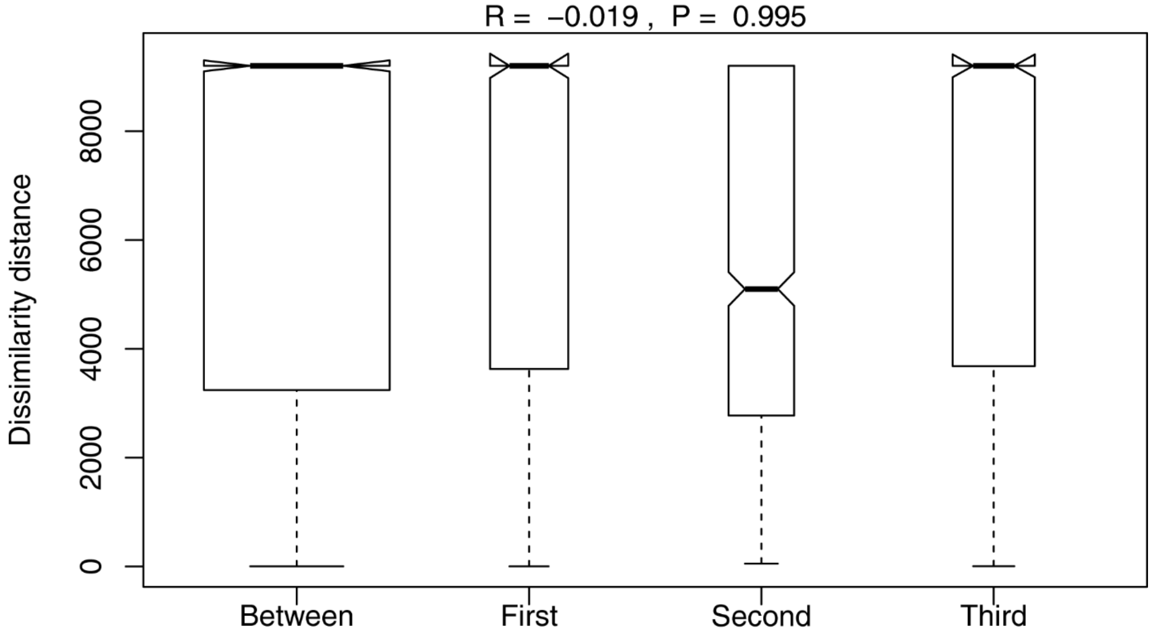

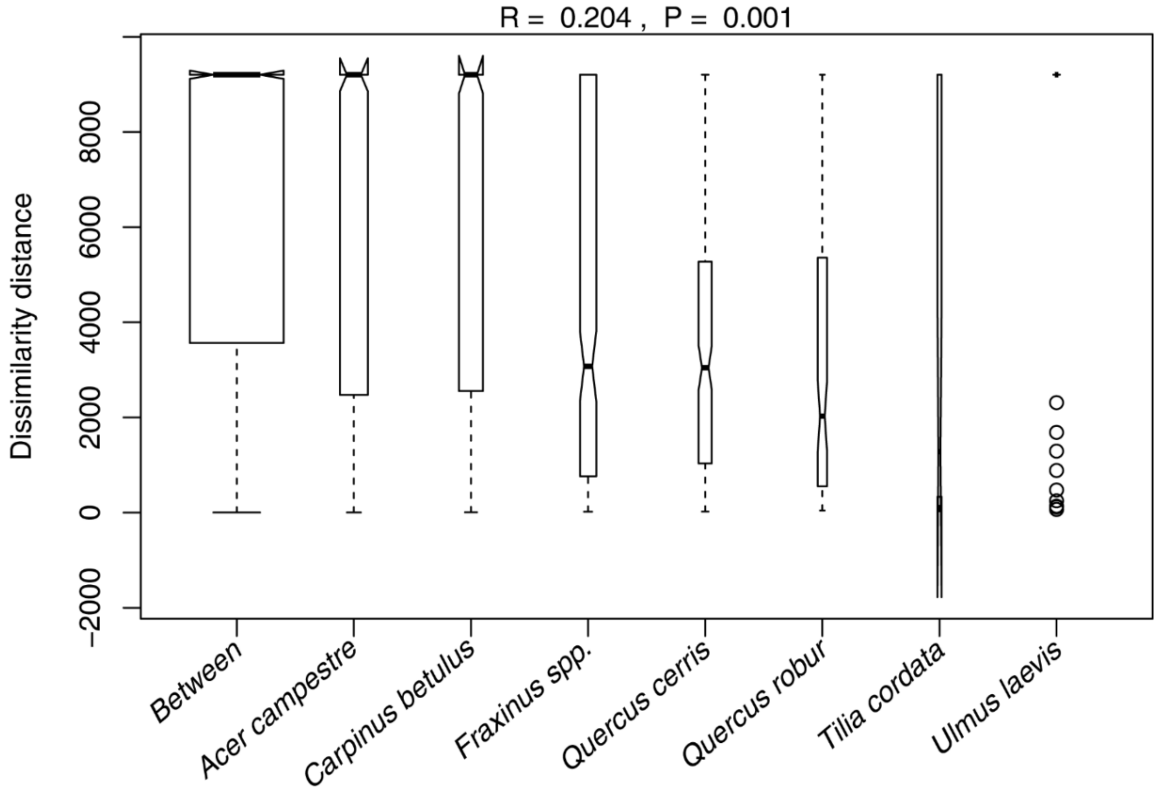

(b)

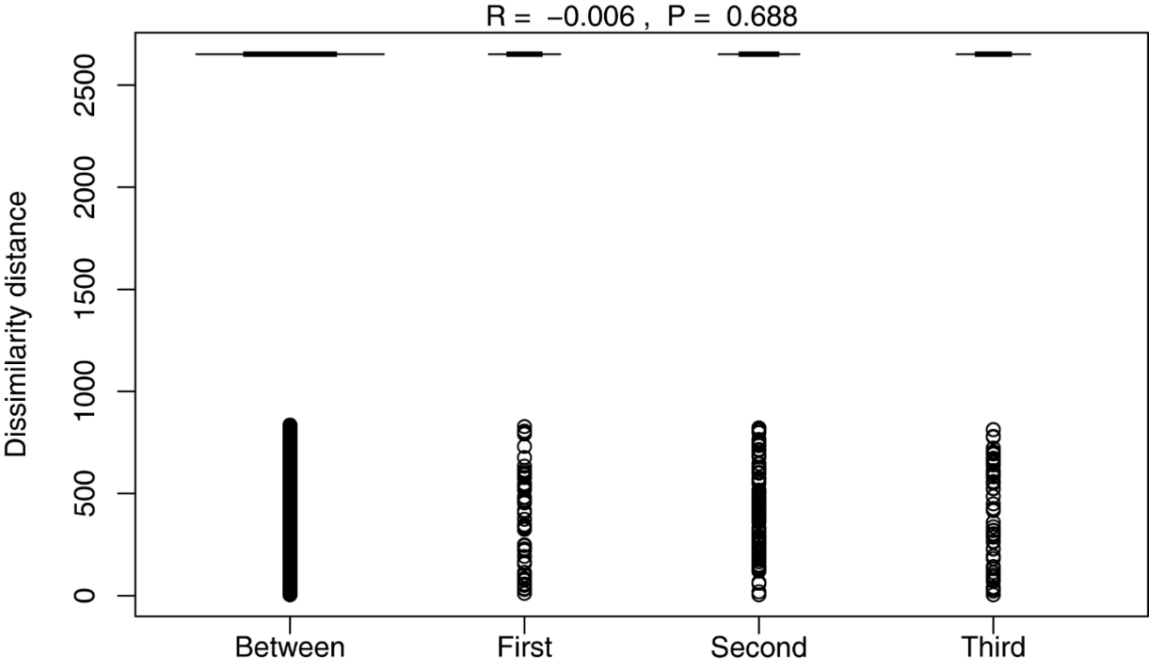

R = −0.006 , P = 0.688
Dissimilarity distance
0
500
1000
1500
2000
2500
Between
First
Second
Third

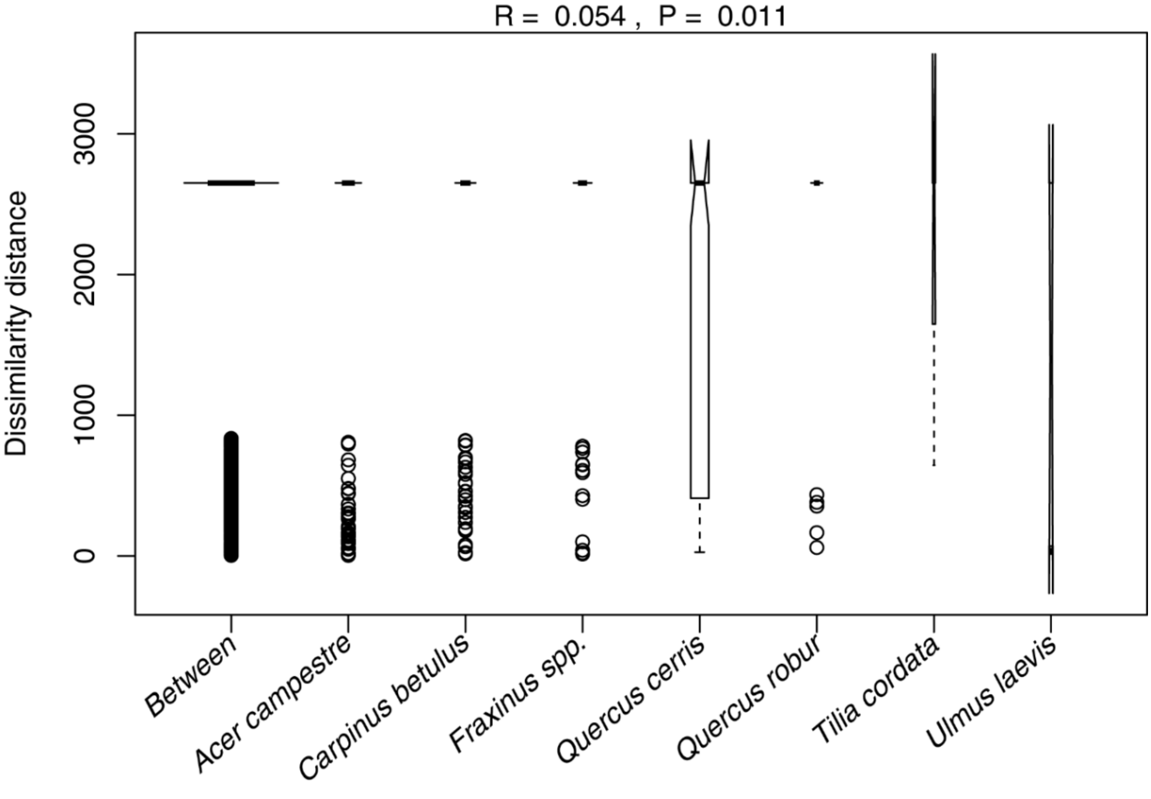

R = 0.054 , P = 0.011
Dissimilarity distance
0
1000
2000
3000
Between
Acer campestre
Carpinus betulus
Fraxinus spp.
Quercus cerris
Quercus robur
Tilia cordata
Ulmus laevis
