# Supplementary material for: Vertical canopy gradient shaping the stratification of leaf‐chewer–parasitoid interactions in a temperate forest
Source: Ecol Evol. 2018 Jun 27;8(15):7297–311. doi: 10.1002/ece3.4194 (PMC6106176; doi:10.1002/ece3.4194)
Supplement: Supplementary file 4 [file ECE3-8-7297-s004.pdf]

Figure S4. Significant variables [(a) canopy levels, (b) tree height, and (c) tree species] tested in likelihood-ratio (LR) analysis of deviance for individual food web indices. Median values, interquantile ranges and total ranges are shown in boxplots. Solid lines represent values fitted by generalized linear model (GLM), and shaded area (shown in scatterplots) 95% confidence intervals. On the y-axis, predicted values of GLM models for respective indices are shown.

(a)

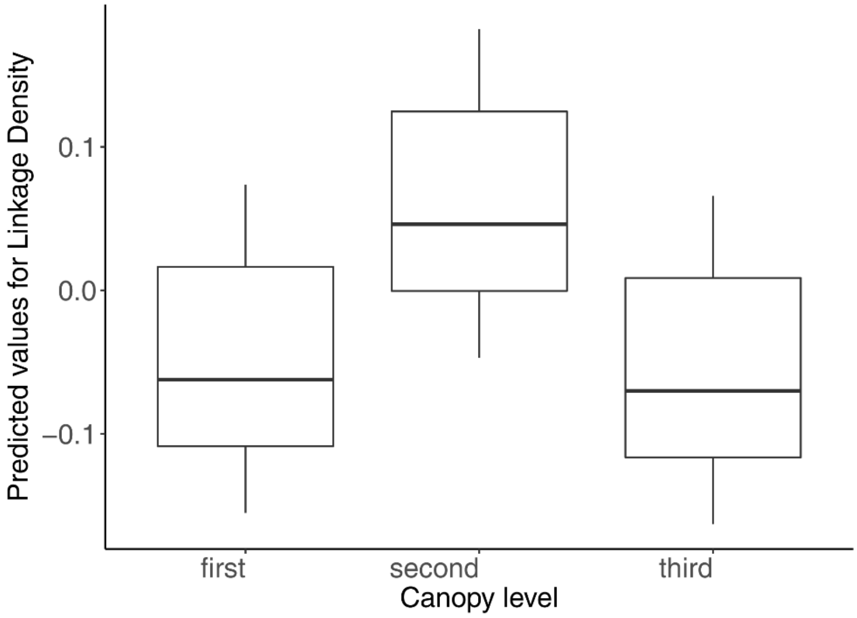

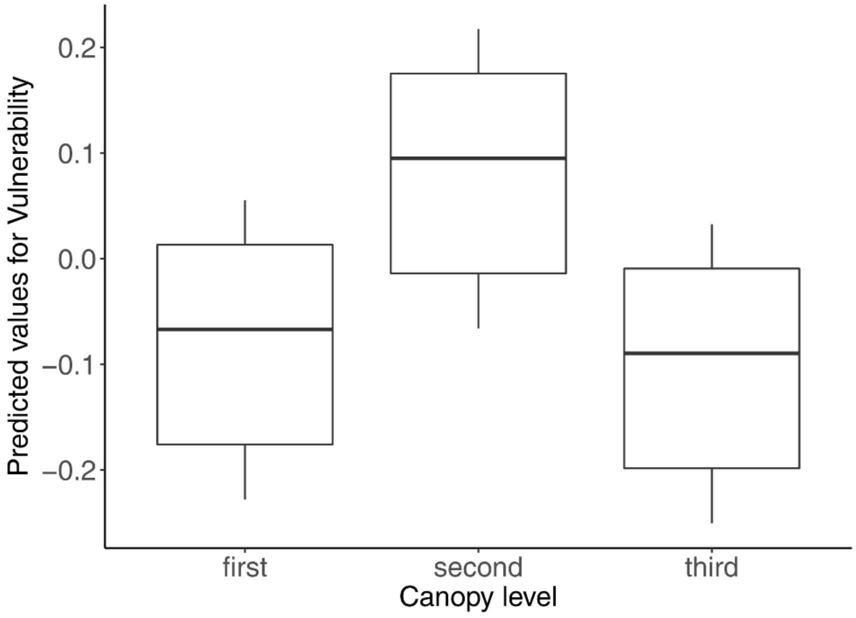

(b)

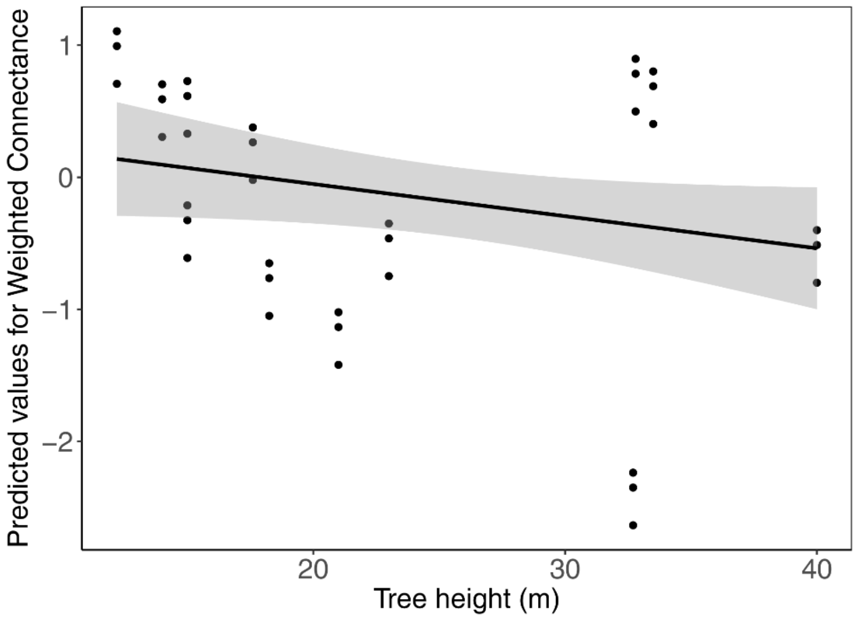

Predicted values for Weighted Connectance
1
0
−1
−2
20
30
40
Tree height (m)

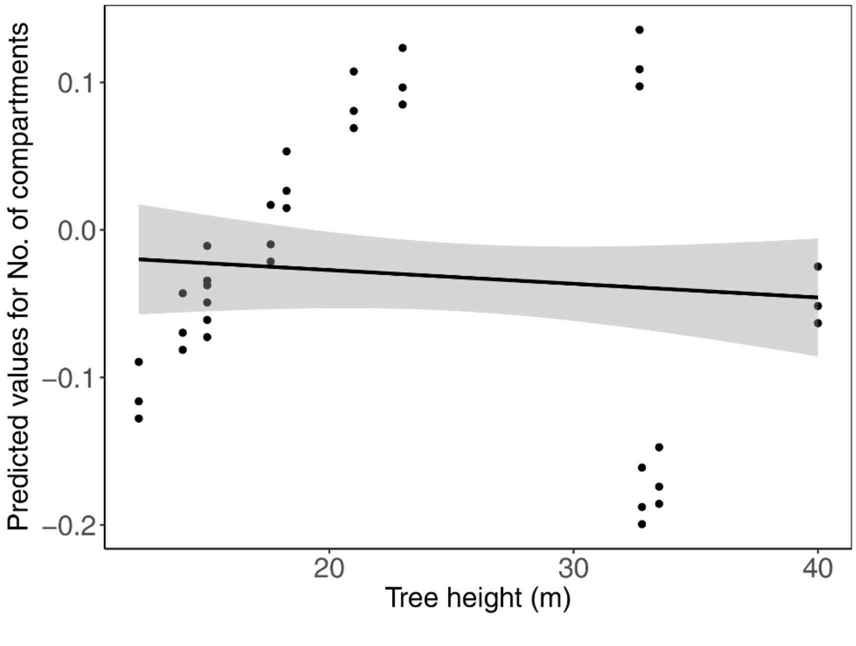

Predicted values for No. of compartments
0.1
0.0
−0.1
−0.2
20
30
40
Tree height (m)

(c)

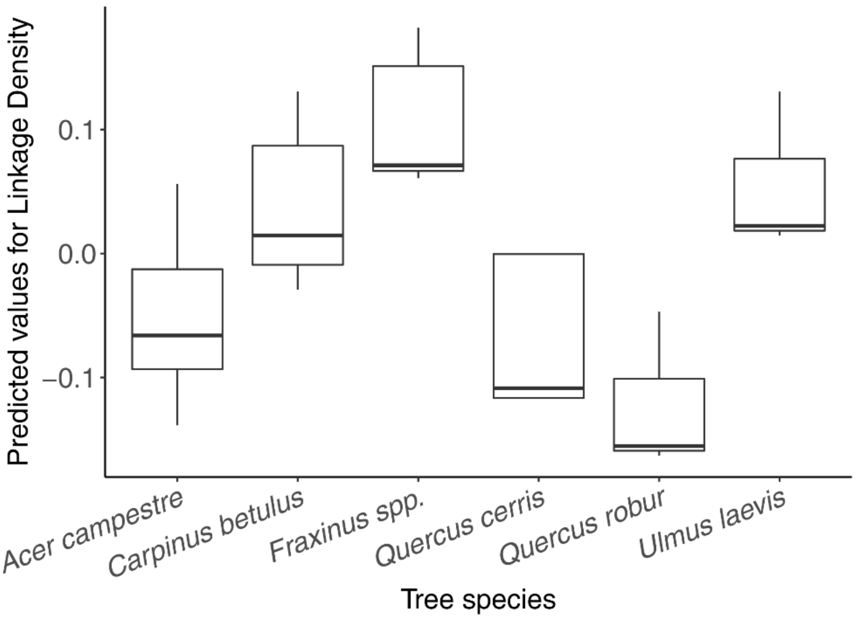

Predicted values for Linkage Density
0.1
0.0
−0.1
Acer campestre
Carpinus betulus
Fraxinus spp.
Quercus cerris
Quercus robur
Ulmus laevis
Tree species

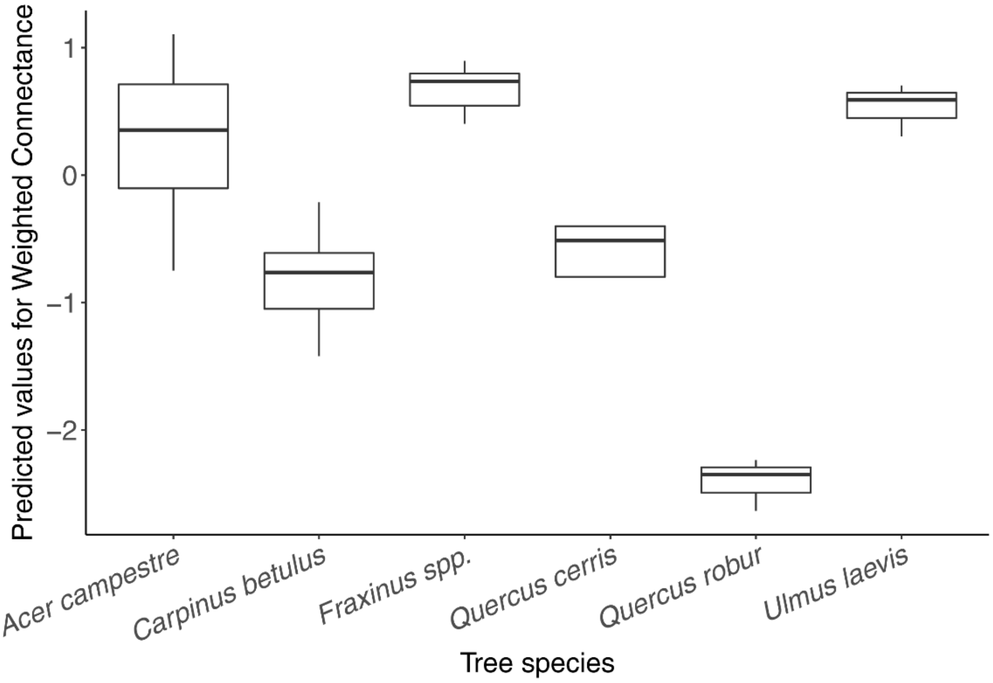

Predicted values for Weighted Connectance
1
0
−1
−2
Acer campestre
Carpinus betulus
Fraxinus spp.
Quercus cerris
Quercus robur
Ulmus laevis
Tree species

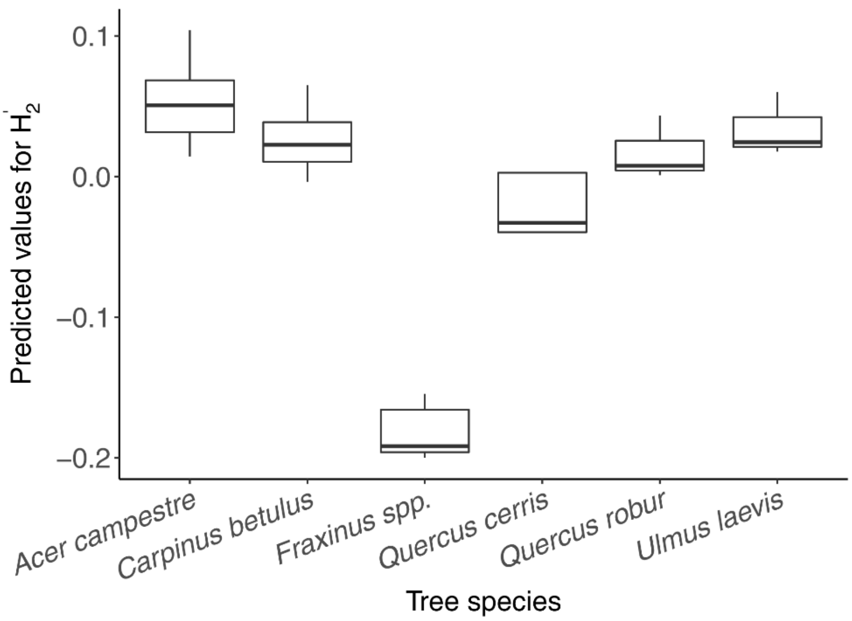

0.1
0.0
−0.1
−0.2
Predicted values for H'2
Acer campestre
Carpinus betulus
Fraxinus spp.
Quercus cerris
Quercus robur
Ulmus laevis
Tree species

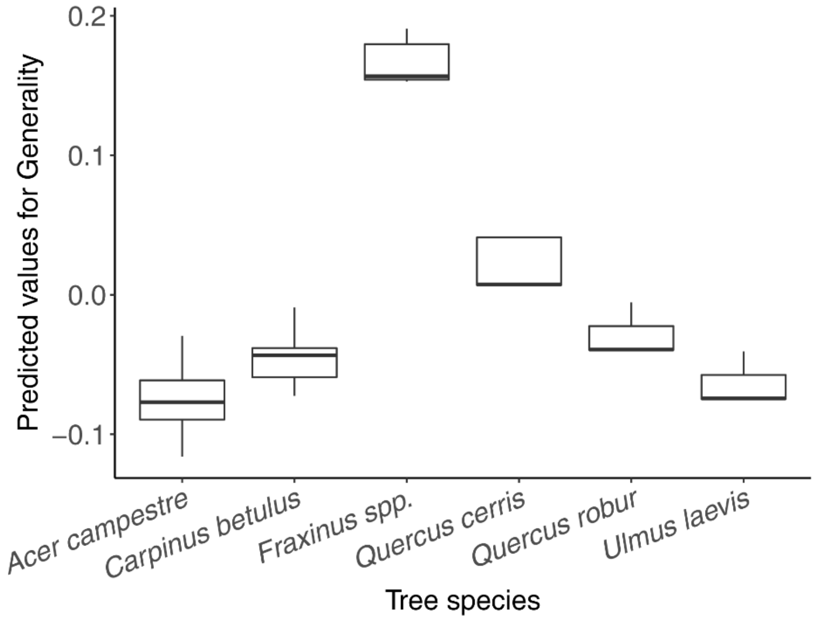

0.2
0.1
0.0
−0.1
Predicted values for Generality
Acer campestre
Carpinus betulus
Fraxinus spp.
Quercus cerris
Quercus robur
Ulmus laevis
Tree species

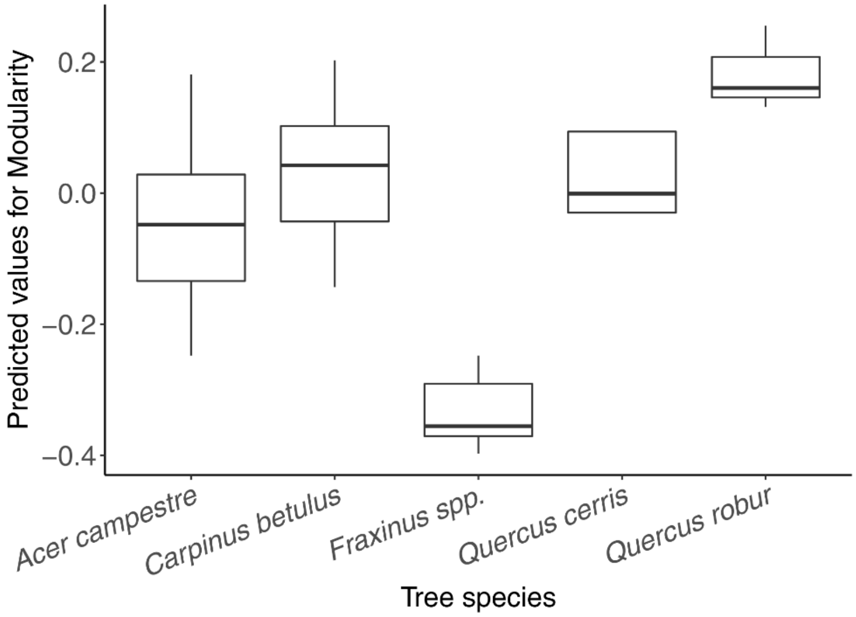
Predicted values for Modularity
0.2
0.0
−0.2
−0.4
Acer campestre
Carpinus betulus
Fraxinus spp.
Quercus cerris
Quercus robur
Tree species

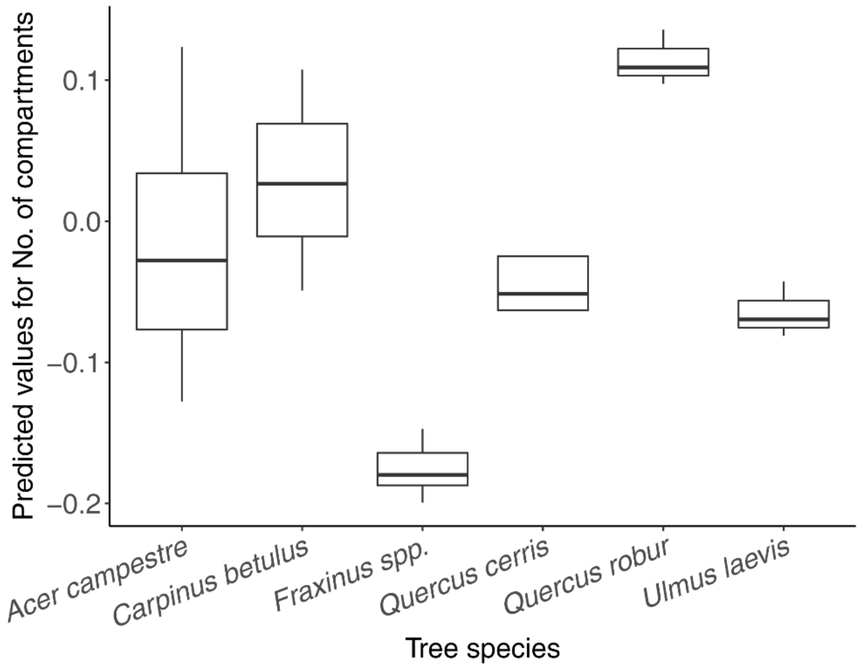
Predicted values for No. of compartments
0.1
0.0
−0.1
−0.2
Acer campestre
Carpinus betulus
Fraxinus spp.
Quercus cerris
Quercus robur
Ulmus laevis
Tree species
