# Supplementary material for: Vertical canopy gradient shaping the stratification of leaf‐chewer–parasitoid interactions in a temperate forest
Source: Ecol Evol. 2018 Jun 27;8(15):7297–311. doi: 10.1002/ece3.4194 (PMC6106176; doi:10.1002/ece3.4194)

Figure S5. Quantitative host–parasitoid food webs among individual tree species. For each web, lower bars represent host abundance (black – parasitized, white – unparasitized), and upper bars parasitoid abundance. Linkage width indicates frequency of each trophic interaction. Webs show interaction data pooled across all replicates for each tree species, although food web indices were calculated for each canopy level of 14 tree individuals (42 networks). Species codes are given in Tables S2 and S3, Supporting Information. The webs are drawn at different scales (Bar width is proportional to species density). For *A. campestre*, *C. betulus*, *Fraxinus* spp., *Q. cerris*, *Q. robur* and *U. laevis* the host abundances are 202, 56, 38, 409, 11 and 28, respectively.

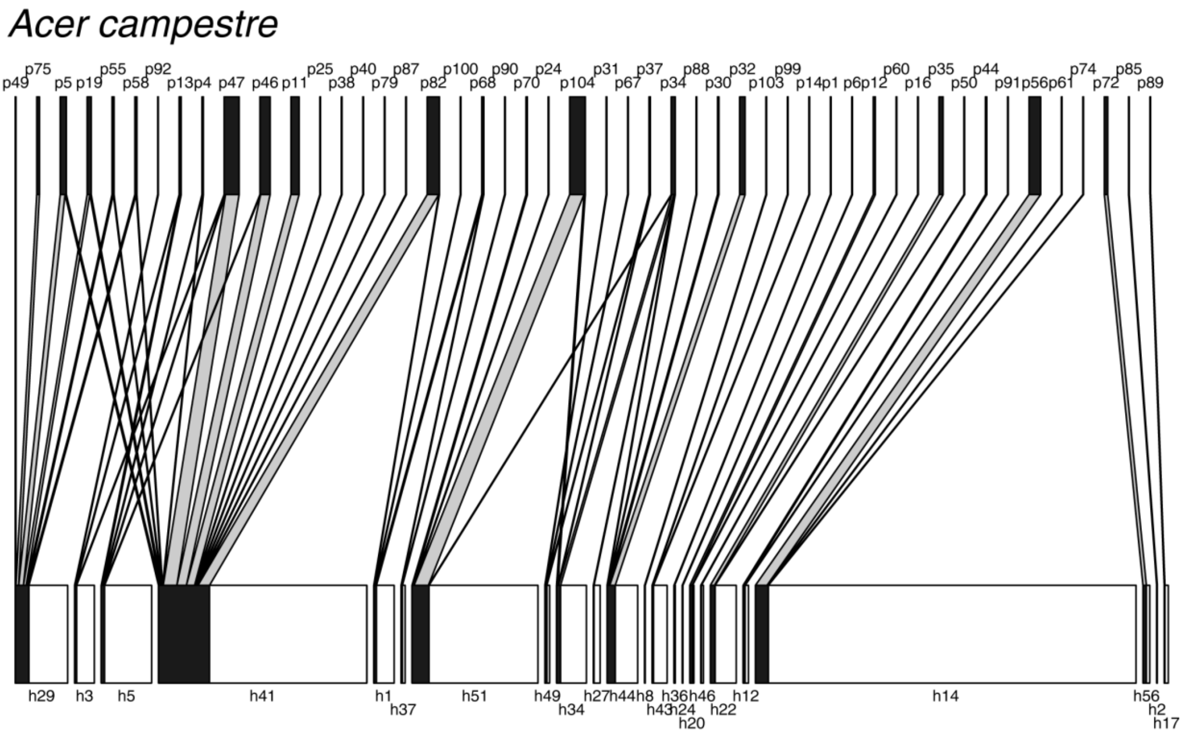

Carpinus betulus

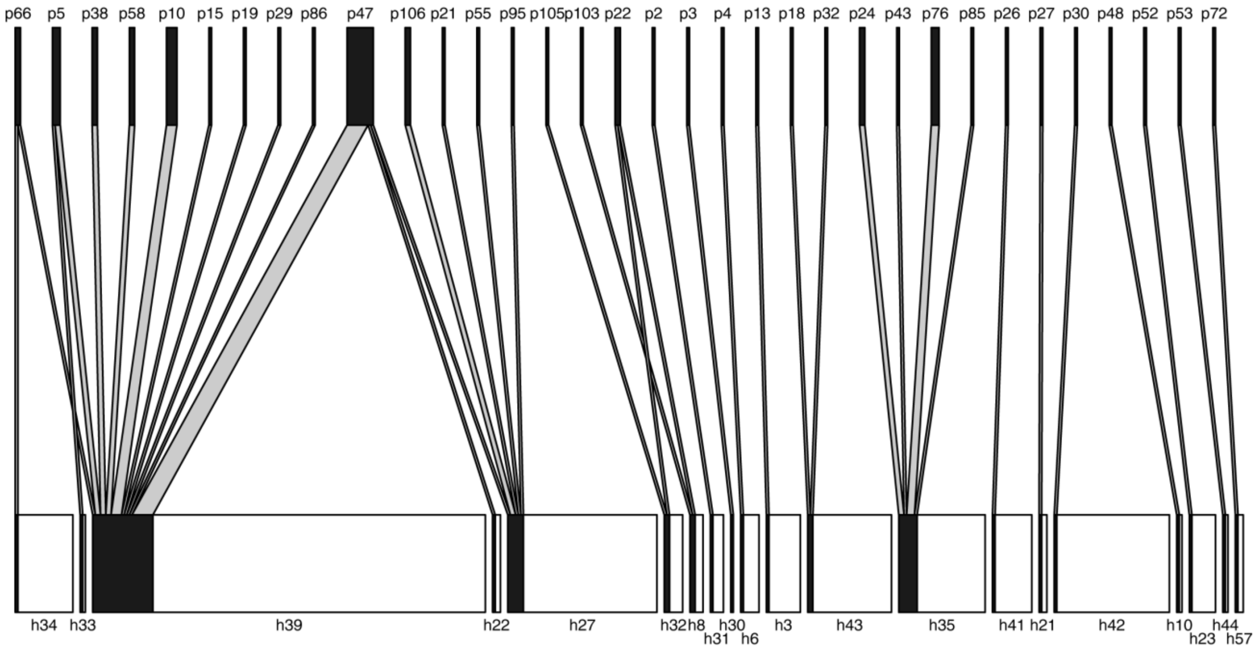

Fraxinus spp.

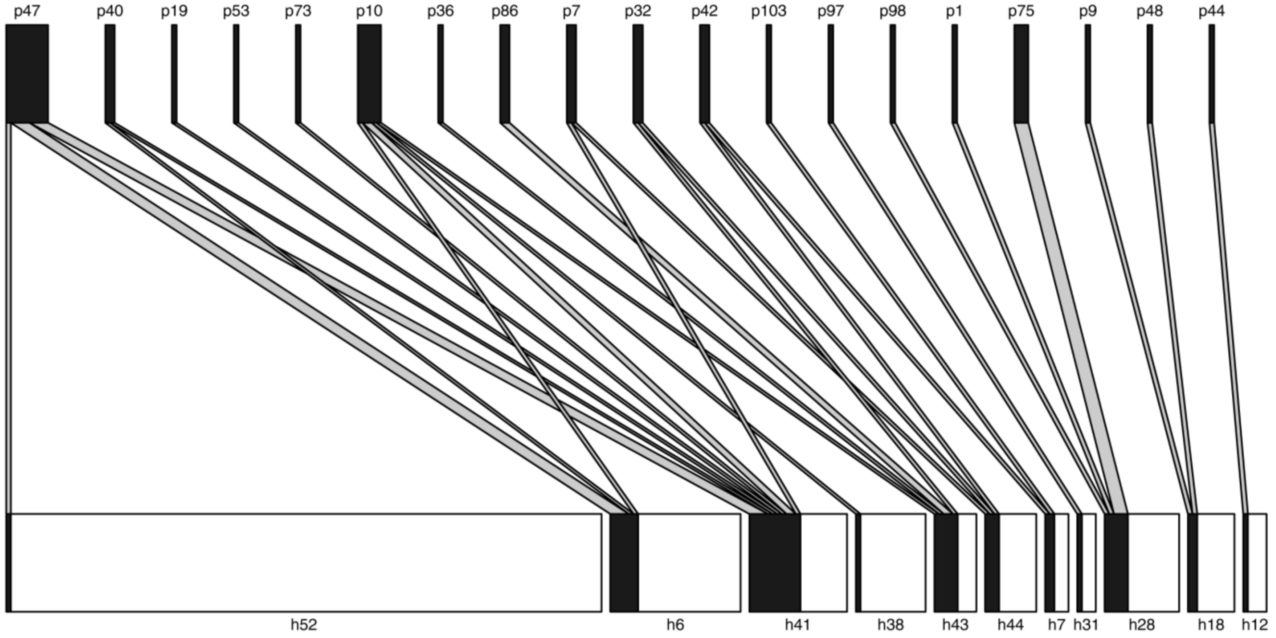

Quercus cerris

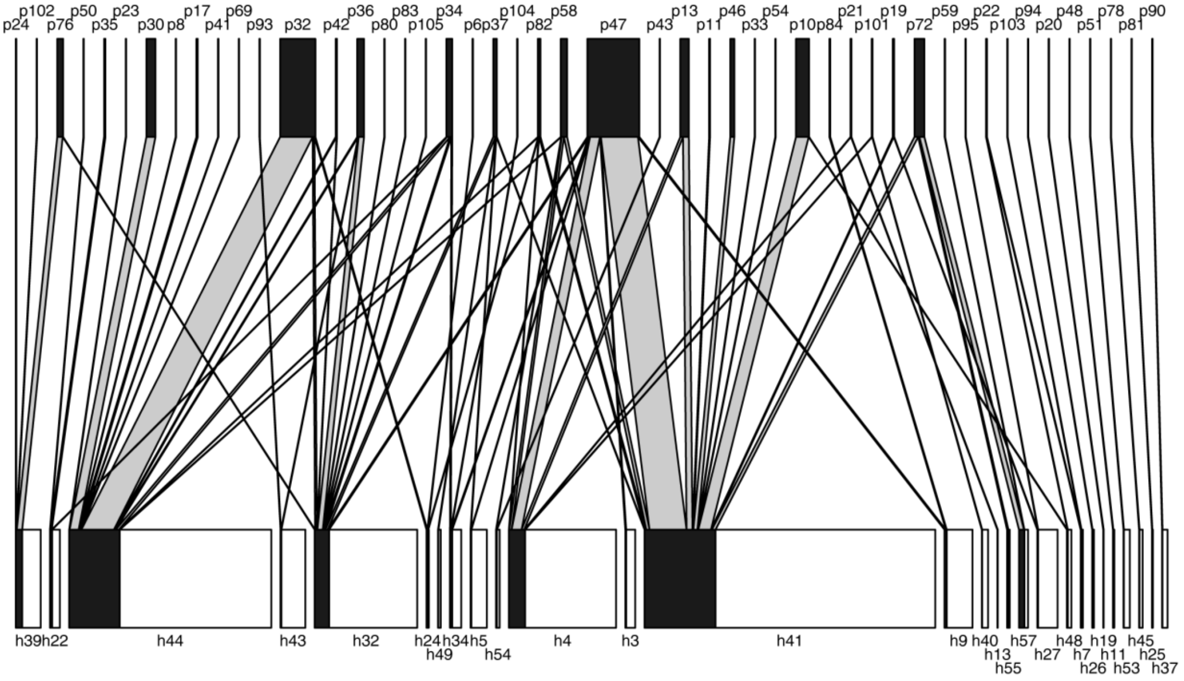

Quercus robur

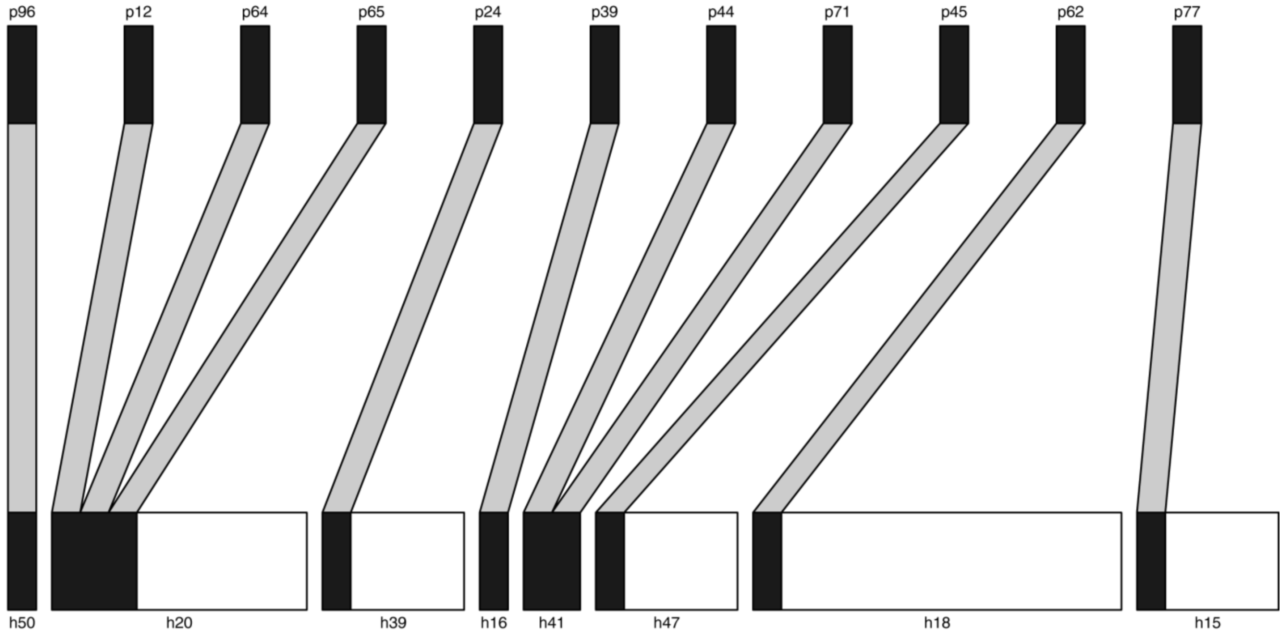

Ulmus laevis

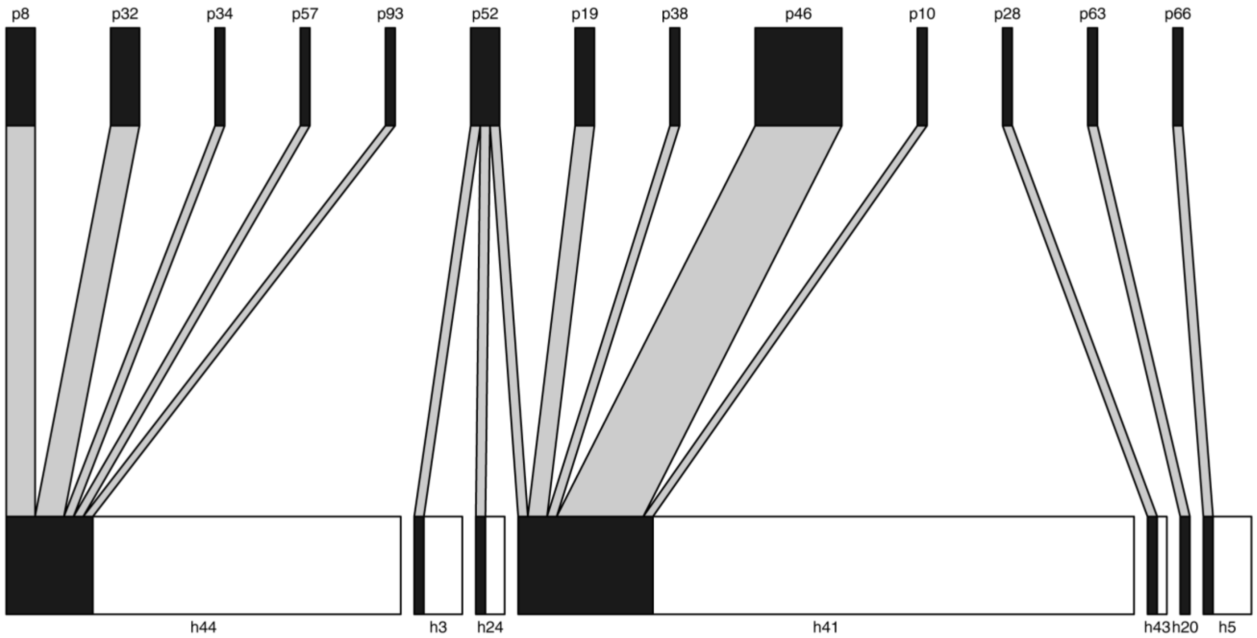

Supplement: Supplementary file 5 [file ECE3-8-7297-s005.pdf]
