# Supplementary material for: Vertical canopy gradient shaping the stratification of leaf‐chewer–parasitoid interactions in a temperate forest
Source: Ecol Evol. 2018 Jun 27;8(15):7297–311. doi: 10.1002/ece3.4194 (PMC6106176; doi:10.1002/ece3.4194)
Supplement: Supplementary file 6 [file ECE3-8-7297-s006.pdf]

**Table S1.** List of taxonomic resources used for identification of host species and parasitoid families.

**Hosts**


De Prins, J., De Prins, W. (2012) Global Taxonomic Database of Gracillariidae (Lepidoptera). URL http://www.gracillariidae.net.

Lacourt, J. (1999) *Répertoire des Tenthredinidae ouest-paléarctiques (Hymenoptera, Symphyta).* Société entomologique de France.

Laštůvka, Z. & Liška, J. (2010) Checklist of Lepidoptera of the Czech Republic (Insecta: Lepidoptera). URL http://lepidoptera.wz.cz.

Macek, J., Dvořák, J., Traxler, L. & Červenka, V. (2007) *Noční motýli I.* Academia, Praha.

Macek, J., Dvořák, J., Traxler, L. & Červenka, V. (2008) *Noční motýli II.* Academia, Praha.

Macek, J., Procházka, J. & Traxler, L. (2012) *Noční motýli III.* Academia, Praha.

Patočka, J. (1980) *Die Raupen und Puppen der Eichenschmetterlinge Mitteleuropas.* Monographien zur angewandten Entomologie.

Razowski, J. (2008) *Tortricidae of the Palaearctic Region, Volume 1 Tortricini and general part.* František Slamka. Bratislava, Kraków.

Razowski, J. (2009) *Tortricidae of the Palaearctic Region, Volume 2, Cochylini.* Frantisek Slamka, Bratislava, 1–195.

Slamka, F. (2010) *Pyraloidea (Lepidoptera) of Central Europe: identification, distribution, habitat, biology.* Frantisek Slamka, Bratislava.

Taeger, A. & Blank, S.M. (1998) *Pflanzenwespen Deutschlands: (Hymenoptera, Symphyta): kommentierte Bestandsaufnahme.* Goecke & Evers.

Tokár, Z., Lvovsky, A., Huemer, P. & Slamka, F. (2005) *Die Oecophoridae sl (Lepidoptera) Mitteleuropas: Bestimmung-Verbreitung-Habitat: Bionomie.* Frantisek Slamka, Bratislava.


**Parasitoids**


Austin, A.D. & Dangerfield, P.C. (1992) Synopsis of Australasian Microgastrinae (Hymenoptera: Braconidae), with a key to genera and description of new taxa. *Invertebrate Taxonomy,* **6,** 1–76.

Bouček, Z. & Rasplus, J.-Y. (1991) *Illustrated Key to West-Palearctic Genera of Pteromalidae (Hymenoptera: Chalcidoidea).* Institut National de la Recherche Agronomique, Paris.

Broad, G. (2011) *Identification Key to the Subfamilies of Ichneumonidae (Hymenoptera).* The Natural History Museum, London, UK.

Dzhanokmen, K.A. (2001) A review of pteromalids of the genus *Pteromalus* Swederus (Hymenoptera, Pteromalidae) of Kazakhstan. II. *Entomologicheskoe Obozrenie,* **80,** 472–496.

Gibson, G.A.P., Huber, J.T. & Woolley, J.B. (1997) *Annotated keys to the genera of Nearctic Chalcidoidea (Hymenoptera).* NRC Research Press, Ottawa.

Goulet, H. & Huber, J.T. (1993) *Hymenoptera of the world: an identification guide to families.* Research Branch, Agriculture Canada.

Graham, M.W.R.D.V. (1969) The Pteromalidae of North-Western Europe (Hymenoptera: Chalcidoidea). *Bulletin of the British Museum (Natural History) Entomology,* **16,** 1–908.

Hansson, C., Smith, M.A., Janzen, D.H. & Hallwachs, W. (2015) Integrative taxonomy of New World *Euplectrus* Westwood (Hymenoptera, Eulophidae), with focus on 55 new species from Area de Conservación Guanacaste, northwestern Costa Rica. *Zookeys,* **485,** 1–236.

Herting, B. (1984) Catalogue of Palearctic Tachinidae (Diptera). *Stuttgarter Beitraege zur Naturkunde. Serie A. Biologie,* **369,** 1–228.

Horstmann, K. (1970) Bemerkungen zur Systematik einiger Gattungen der Campopleginae (Hymenoptera, Ichneumonidae). *Nachrichtenblatt der Bayerischen Entomologen,* **19,** 77–84.

Horstmann, K. (1977) Bemerkungen zur Systematik einiger Gattungen der Campopleginae II (Hymenoptera, Ichneumonidae). *Mitteilungen der Münchner Entomologischen Gesellschaft,* **67,** 65–83.

Horstmann, K. (1986) Bemerkungen zur Systematik einiger Gattungen der Campopleginae III (Hymenoptera, Ichneumonidae). *Mitteilungen der Münchner Entomologischen Gesellschaft,* **76,** 143–164.

Horstmann, K. (2004) Bemerkungen zur Systematik einiger Gattungen der Campopleginae IV (Hymenoptera, Ichneumonidae). *Zeitschrift der Arbeitsgemeinschaft Österreichischer Entomologen,* **56,** 13–35.

Chvála, M. (1997) *Check list of Diptera (Insecta) of the Czech and Slovak Republics*. Karolinum, Charles University Press.

Kalina, V. (1989) Checklist of Czechoslovak Insects III (Hymenoptera). Chalcidoidea. *Acta Faunistica Entomologica Musei Nationalis Pragae,* **19,** 97–127.

Malloch, J.R. (1917) A preliminary classification of Diptera, exclusive of Pupipara, based upon larval and pupal characters, with keys to imagines in certain families. *Illinois Natural History Survey Bulletin,* **12,** 161–409.

Noyes, J.S. (2016) Universal Chalcidoidea Database. World Wide Web electronic publication.

Peck, O., Bouček, Z. & Hoffer, A. (1964) Keys to the Chalcidoidea of Czechoslovakia (Insecta: Hymenoptera). *Memoirs of the Entomological Society of Canada,* **96,** 7–121.

Perkins, J.F. (1959) Ichneumonidae, key to subfamilies and Ichneumoninae - 1. *Handbooks for the Identification of British Insects,* **7,** 1–116.

Sabrosky, C.W. & Reardon, R.C. (1976) Tachinid parasites of the gypsy moth, *Lymantria dispar*, with keys to adults and puparia. *Miscellaneous Publications of the Entomological Society of America,* **10,** 1–126.

Shaw, M.R. & Huddleston, T. (1991) Classification and biology of braconid wasps (Hymenoptera: Braconidae). *Handbooks for the Identification of British Insects,* **7,** 1–126.

Schauff, M.E. & Janzen, D.H. (2001) Taxonomy and ecology of Costa Rican Euplectrus (Hymenoptera: Eulophidae), parasitoids of caterpillars. *Journal of Hymenoptera Research,* **10,** 181–230.

Sureshan, P.M. (2001) Studies on *Pteromalus* Swederus (Hymenoptera: Chalcidoidea: Pteromalidae) of the Indian subcontinent with the description of three new species. *Records of the Zoological Survey of India,* **99,** 5–14.

Šedivý, J. (1989) Checklist of Czechoslovak Insects III (Hymenoptera). *Acta Faunistica Entomologica Musei Nationalis Pragae,* **19,** 1–194.

Tschorsnig, H.-P. & Herting, B. (1994) The tachinids (Diptera: Tachinidae) of Central Europe: identification keys for the species and data on distribution and ecology. *Stuttgarter Beitraege zur Naturkunde. Serie A. Biologie,* **506,** 1–170.

Van Achterberg, C. (1990) Illustrated key to the subfamilies of the Holarctic Braconidae (Hymenoptera: Ichneumonoidea). *Zoologische Mededelingen,* **64,** 1–20.

Van Achterberg, C. (2013) Fauna Europaea – Ichneumonoidea. URL http://faunaeur.org.

Vaňhara, J., Tschorsnig, H.-P., Herting, B., Mückstein, P. & Michalková, V. (2009) Annotated host catalogue for the Tachinidae (Diptera) of the Czech Republic. *Entomologica Fennica,* **20,** 22–48.

Venkatesha, M.G. & Gopinath, K. (1994) Description of immature stages of a species of Glyptapanteles (Hymenoptera: Braconidae), a gregarious endoparasitoid of Amata passalis (Fabricius) (Lepidoptera: Arctiidae), a defoliator of sandalwood, Santalum album L. *Insect Science and Its Application,* **15,** 161–165.

Yu, D.S., van Achterberg, C. & Horstmann, K. (2012) Home of Ichneumonoidea. URL http://www.taxapad.com.

Zhu, C.-D. & Huang, D.-W. (2003) A study of the genus *Euplectrus* Westwood (Hymenoptera: Eulophidae) in China. *Zoological Studies,* **42,** 140–164.
