# Supplementary material for: Vertical canopy gradient shaping the stratification of leaf‐chewer–parasitoid interactions in a temperate forest
Source: Ecol Evol. 2018 Jun 27;8(15):7297–311. doi: 10.1002/ece3.4194 (PMC6106176; doi:10.1002/ece3.4194)
Supplement: Supplementary file 7 [file ECE3-8-7297-s007.pdf]

Table S2. List of parasitoids. BIN = Barcoding Index Number (putative species); n = number of parasitoid rearing events; Host feeding mode = host guild exploited by particular parasitoid species; PD = host phylogenetic diversity for particular parasitoid species (Faith, 1992; Poulin et al., 2011) calculated as a sum of all branch lengths connecting the focal set of its host species in a phylogenetic tree (Figure S1, Supporting Information) and divided by the number of host species (parasitoids exploiting three of more individuals of the same species and no other species were considered as monophagous and their PD values were set to zero, while single- and doubletons were excluded from the analysis); S = number of host species; c = code of respective species used in host–parasitoid food webs for individual tree species (Figure S5, Supporting Information).

Family	BIN*	n	Host feeding mode	PD	S	c
Braconidae	BOLD:AAA3092	3	exposed	22.0	2	p1
Braconidae	BOLD:AAA5826	6	semi-concealed	31.8	4	p2
Braconidae	BOLD:AAA7148	1	exposed	NA	1	p3
Braconidae	BOLD:AAA7151	5	exposed	26.5	4	p4
Braconidae	BOLD:AAA7795	16	exposed	49.8	4	p5
Braconidae	BOLD:AAA7796	5	both	20.0	3	p6
Braconidae	BOLD:AAA9379	2	exposed	138.0	2	p7
Braconidae	BOLD:AAB1922	1	semi-concealed	NA	1	
Tachinidae	BOLD:AAC1602	4	exposed	0.0	1	p8
Braconidae	BOLD:AAE8368	3	both	98.0	2	p9
Tachinidae	BOLD:AAF6259	66	exposed	69.4	5	p10
Ichneumonidae	BOLD:AAG8326	14	exposed	0.0	1	p11
Ichneumonidae	BOLD:AAG8409	1	semi-concealed	NA	1	
Braconidae	BOLD:AAH1360	6	semi-concealed	0.0	1	p12
Ichneumonidae	BOLD:AAI1095	29	exposed	14.3	4	p13
Braconidae	BOLD:AAI1657	3	semi-concealed	NA	1	
Ichneumonidae	BOLD:AAI3096	1	exposed	NA	1	p14
Braconidae	BOLD:AAI4821	10	semi-concealed	10.0	2	
Ichneumonidae	BOLD:AAI5646	2	both	98.0	2	p15
Ichneumonidae	BOLD:AAL8261	1	semi-concealed	NA	1	p16
Tachinidae	BOLD:AAN4017	3	exposed	0.0	1	p17
Tachinidae	BOLD:AAN4077	1	exposed	NA	1	p18
Ichneumonidae	BOLD:AAU8361	15	exposed	94.0	4	p19
Braconidae	BOLD:AAV0367	1	semi-concealed	NA	1	p20
Tachinidae	BOLD:AAV0903	3	exposed	95.0	3	p21
Tachinidae	BOLD:AAV0908	4	exposed	13.0	4	p22
Braconidae	BOLD:AAV2050	1	exposed	NA	1	p23
Braconidae	BOLD:AAV2164	6	exposed	98.0	2	p24
Ichneumonidae	BOLD:AAW9099	1	exposed	NA	1	p25
Tachinidae	BOLD:AAZ5302	2	exposed	NA	1	p26
Braconidae	BOLD:AAZ9567	1	semi-concealed	NA	1	p27
Ichneumonidae	BOLD:AAZ9885	1	exposed	NA	1	p28
Ichneumonidae	BOLD:ABA8712	1	NA	NA	NA	
Ichneumonidae	BOLD:ABU6867	1	exposed	NA	1	p29
Braconidae	BOLD:ABU7097	1	NA	NA	NA	
Ichneumonidae	BOLD:ABV4816	23	exposed	5.0	2	p30
Ichneumonidae	BOLD:ABV5256	2	exposed	NA	1	p31
Braconidae	BOLD:ABV9098	98	exposed	9.2	5	p32

Braconidae	BOLD:ABY2372	3	exposed	NA	1	p33
Ichneumonidae	BOLD:ACA3906	1	semi-concealed	NA	1	
Tachinidae	BOLD:ACB1072	28	exposed	41.5	10	p34
Tachinidae	BOLD:ACD2706	10	exposed	0.0	1	p35
Braconidae	BOLD:ACD4863	19	exposed	5.7	3	p36
Tachinidae	BOLD:ACD9465	11	exposed	64.6	5	p37
Braconidae	BOLD:ACE8790	1	NA	NA	NA	
Braconidae	BOLD:ACE9542	8	exposed	0.0	1	p38
Ichneumonidae	BOLD:ACG0304	2	both	175.0	2	p39
Ichneumonidae	BOLD:ACG4708	5	semi-concealed	0.0	1	
Ichneumonidae	BOLD:ACG5352	2	both	140.0	2	
Braconidae	BOLD:ACI0522	6	exposed	35.0	3	p40
Ichneumonidae	BOLD:ACJ1142	1	exposed	NA	1	p41
Eulophidae	BOLD:ACK1468	5	exposed	16.7	3	p42
Braconidae	BOLD:ACL7229	3	both	111.3	3	p43
Ichneumonidae	BOLD:ACL8803	5	both	67.7	3	p44
Ichneumonidae	BOLD:ACL9499	2	both	98.0	2	p45
Tachinidae	BOLD:ACM9631	67	exposed	27.0	2	p46
Braconidae	BOLD:ACO3086	1	exposed	NA	1	
Tachinidae	BOLD:ACO3982	1	exposed	NA	1	
Tachinidae	BOLD:ACO3995	254	both	57.6	12	p47
Braconidae	BOLD:ACQ1836	4	both	124.7	3	p48
Ichneumonidae	BOLD:ACR0021	1	exposed	NA	1	p49
Ichneumonidae	BOLD:ACR0208	2	semi-concealed	10.0	2	
Tachinidae	BOLD:ACR1744	2	exposed	NA	1	p50
Ichneumonidae	BOLD:ACR2948	3	semi-concealed	0.0	1	p51
Ichneumonidae	BOLD:ACR4279	4	exposed	76.0	4	p52
Ichneumonidae	BOLD:ACR4523	3	exposed	138.0	2	p53
Eulophidae	BOLD:ACR7504	7	exposed	14.0	4	p54
Eulophidae	BOLD:ACR7505	6	exposed	94.0	4	p55
Ichneumonidae	BOLD:ACT9980	17	both	0.0	1	p56
Eulophidae	BOLD:ACU2768	1	exposed	NA	1	p57
Braconidae	BOLD:ACU2814	20	both	76.8	6	p58
Ichneumonidae	BOLD:ACU2904	1	semi-concealed	NA	1	p59
Eulophidae	BOLD:ACU2918	5	exposed	15.0	2	
Eulophidae	BOLD:ACU2970	1	semi-concealed	NA	1	p60
Ichneumonidae	BOLD:ACU3041	2	both	NA	1	p61
Eulophidae	BOLD:ACU3102	2	exposed	15.0	2	p62
Eulophidae	BOLD:ACU3127	1	semi-concealed	NA	1	p63
Braconidae	BOLD:ACU3184	1	exposed	NA	1	
Eulophidae	BOLD:ACU3213	1	semi-concealed	NA	1	p64
Ichneumonidae	BOLD:ACU3222	1	semi-concealed	NA	1	p65
Eulophidae	BOLD:ACU3230	34	exposed	12.7	6	p66
Eulophidae	BOLD:ACU3231	3	exposed	5.0	2	p67
Eulophidae	BOLD:ACU3259	1	exposed	NA	1	
Ichneumonidae	BOLD:ACU3261	3	semi-concealed	27.0	2	p68
Braconidae	BOLD:ACU3265	1	exposed	NA	1	p69
Eulophidae	BOLD:ACU3276	2	exposed	NA	1	p70

Ichneumonidae	BOLD:ACU3357	1	exposed	NA	1	p71
Ichneumonidae	BOLD:ACU3358	28	both	88.8	4	p72
Braconidae	BOLD:ACU3556	4	semi-concealed	3.0	2	
Ichneumonidae	BOLD:ACU3578	3	both	147.0	2	p73
Eulophidae	BOLD:ACU3590	1	both	NA	1	p74
Eulophidae	BOLD:ACU3754	1	NA	NA	NA	
Ichneumonidae	BOLD:ACU3760	2	NA	NA	NA	
Eulophidae	BOLD:ACU3777	1	NA	NA	NA	
Ichneumonidae	BOLD:ACU3853	7	exposed	138.0	2	p75
Ichneumonidae	BOLD:ACU3901	25	exposed	104.0	3	p76
Braconidae	BOLD:ACU3911	1	both	NA	1	p77
Ichneumonidae	BOLD:ACU3912	1	semi-concealed	NA	1	p78
Braconidae	BOLD:ACU3995	2	semi-concealed	14.0	2	
Braconidae	BOLD:ACU3996	1	semi-concealed	NA	1	
Braconidae	BOLD:ACU3999	1	exposed	NA	1	
Braconidae	BOLD:ACU4000	3	exposed	65.7	3	
Eulophidae	BOLD:ACU4046	1	exposed	NA	1	p79
Braconidae	BOLD:ACV0058	1	exposed	NA	1	p80
Braconidae	BOLD:ACV1043	1	semi-concealed	NA	1	p81
Perilampidae	BOLD:ACV1055	28	both	70.3	6	p82
Ichneumonidae	BOLD:ACV1285	1	exposed	NA	1	p83
Eulophidae	BOLD:ACV2449	1	NA	NA	NA	
Ichneumonidae	BOLD:ACV4204	2	exposed	NA	1	p84
Tachinidae	BOLD:ACX8696	3	exposed	32.7	3	p85
Ichneumonidae	BOLD:ACY7617	3	exposed	138.0	2	p86
Braconidae	BOLD:ACZ1252	1	exposed	NA	1	p87
Tachinidae	BOLD:ACZ8983	1	exposed	NA	1	p88
Braconidae	BOLD:ADA5405	1	semi-concealed	NA	1	p89
Braconidae	BOLD:ADC1631	7	both	85.3	4	p90
Braconidae	BOLD:ADC1632	1	semi-concealed	NA	1	p91
Ichneumonidae	BOLD:ADE2663	2	semi-concealed	38.0	2	
Encyrtidae	BOLD:ADE4700	4	semi-concealed	38.0	2	
Tachinidae	BOLD:ADF0991	1	semi-concealed	NA	1	
Braconidae	BOLD:ADF2469	3	semi-concealed	51.3	3	
Braconidae	BOLD:ADF2471	1	semi-concealed	NA	1	
Tachinidae	BOLD:ACH1961	3	exposed	9.0	2	p92
Braconidae	Brac.sp01	2	exposed	5.0	2	p93
Braconidae	Brac.sp06	1	semi-concealed	NA	1	p94
Braconidae	Brac.sp14	2	exposed	NA	1	p95
unidentified	Dip.sp01	1	semi-concealed	NA	1	
Eulophidae	Eul.sp17	1	semi-concealed	NA	1	p96
unidentified	Hym.sp02	1	semi-concealed	NA	1	
unidentified	Hym.sp03	1	semi-concealed	NA	1	
unidentified	Hym.sp04	1	semi-concealed	NA	1	p97
Ichneumonidae	Ichne.sp05	1	exposed	NA	1	p98
Ichneumonidae	Ichne.sp09	1	exposed	NA	1	p99
Ichneumonidae	Ichne.sp10	1	semi-concealed	NA	1	
Ichneumonidae	Ichne.sp11	1	semi-concealed	NA	1	p100

Ichneumonidae	Ichne.sp12	2	exposed	140.0	2	p101
Ichneumonidae	Ichne.sp13	1	exposed	NA	1	p102
Ichneumonidae	Ichne.sp33	8	exposed	2.0	2	p103
Ichneumonidae	Ichne.sp46	1	semi-concealed	NA	1	
Ichneumonidae	Ichne.sp47	1	semi-concealed	NA	1	
Phoridae	Phor.sp01	1	NA	NA	NA	
Tachinidae	Tach.sp25	24	exposed	39.0	2	p104
Tachinidae	Tach.sp28	2	exposed	NA	1	p105
Tachinidae	Tach.sp34	2	exposed	NA	1	p106
Braconidae	unidentified	13	NA	NA	NA	
Eulophidae	unidentified	3	NA	NA	NA	
Ichneumonidae	unidentified	35	NA	NA	NA	
Tachinidae	unidentified	23	NA	NA	NA	
unidentified	unidentified	70	NA	NA	NA	

* Including 21 distinct morphospecies, which were not successfully barcoded, and unidentified specimens

References:

Faith, D.P. (1992) Conservation evaluation and phylogenetic diversity. *Biological Conservation*, **61**, 1–10.

Poulin, R., Krasnov, B.R. & Mouillot, D. (2011) Host specificity in phylogenetic and geographic space. *Trends in Parasitology*, **27**, 355–361.
