# Supplementary material for: Vertical canopy gradient shaping the stratification of leaf‐chewer–parasitoid interactions in a temperate forest
Source: Ecol Evol. 2018 Jun 27;8(15):7297–311. doi: 10.1002/ece3.4194 (PMC6106176; doi:10.1002/ece3.4194)
Supplement: Supplementary file 8 [file ECE3-8-7297-s008.pdf]

Table S3. List of leaf chewers (hosts). n = number of individuals; c = code of respective species used in host–parasitoid food webs for individual tree species (Figure S5, Supporting Information). Host taxonomy of Lepidoptera and Tenthredinidae follows Laštůvka & Liška (2010), and Lacourt (1999).

Family	Genus	Species	Feeding Mode*	Author(s)	n	c
Tortricidae	*Acleris*	*Acleris ferrugana*	semi-concealed	(Denis & Schiffermüller, 1775)	2	
Tortricidae	*Acleris*	*Acleris forsskaleana*	semi-concealed	(Linnaeus, 1758)	32	h1
Tortricidae	*Acleris*	*Acleris rhombana*	semi-concealed	(Denis & Schiffermüller, 1775)	2	
Tortricidae	*Acleris*	*Acleris sparsana*	semi-concealed	(Denis & Schiffermüller, 1775)	4	
Tortricidae	*Acleris*	*Acleris variegana*	semi-concealed	(Denis & Schiffermüller, 1775)	2	
Noctuidae	*Acronicta*	*Acronicta alni*	exposed	(Linnaeus, 1767)	1	h2
Geometridae	*Agriopis*	*Agriopis aurantiaria*	exposed	(Hübner, 1799)	249	h3
Geometridae	*Agriopis*	*Agriopis leucophaearia*	exposed	(Denis & Schiffermüller, 1775)	247	h4
Geometridae	*Agriopis*	*Agriopis marginaria*	exposed	(Fabricius, 1776)	171	h5
Noctuidae	*Agrochola*	*Agrochola circellaris*	exposed	(Hufnagel, 1766)	1	
Tortricidae	*Aleimma*	*Aleimma loeflingiana*	semi-concealed	(Linnaeus, 1758)	1	
Geometridae	*Alsophila*	*Alsophila aceraria*	exposed	(Denis & Schiffermüller, 1775)	4	
Geometridae	*Alsophila*	*Alsophila aescularia*	exposed	(Denis & Schiffermüller, 1775)	107	h6
Noctuidae	*Amphipyra*	*Amphipyra berbera*	exposed	Rungs, 1949	11	h7
Noctuidae	*Amphipyra*	*Amphipyra pyramidea*	exposed	(Linnaeus, 1758)	13	h8
Gelechiidae	*Anacampsis*	*Anacampsis blattariella*	semi-concealed	(Hübner, 1796)	1	
Gelechiidae	*Anacampsis*	*Anacampsis timidella*	semi-concealed	(Wocke, 1887)	1	
Gelechiidae	*Anarsia*	*Anarsia lineatella*	semi-concealed	Zeller, 1839	1	
Tenthredinidae	*Apethymus*	*Apethymus cereus*	exposed	(Klug, 1818)	71	h9
Tenthredinidae	*Apethymus*	*Apethymus cerris*	exposed	(Kollar, 1850)	6	
Limacodidae	*Apoda*	*Apoda limacodes*	exposed	(Hufnagel, 1766)	6	
Geometridae	*Apocheima*	*Apocheima hispidaria*	exposed	(Denis & Schiffermüller, 1775)	6	
Tortricidae	*Archips*	*Archips crataegana*	semi-concealed	(Hübner, 1799)	1	
Tortricidae	*Archips*	*Archips podana*	semi-concealed	(Scopoli, 1763)	7	h10
Tortricidae	*Archips*	*Archips xylosteana*	semi-concealed	(Linnaeus, 1758)	7	
Geometridae	*Ascotis*	*Ascotis selenaria*	exposed	(Denis & Schiffermüller, 1775)	1	
Noctuidae	*Asteroscopus*	*Asteroscopus sphinx*	exposed	(Hufnagel, 1766)	18	h11
Geometridae	*Asthena*	*Asthena albulata*	exposed	(Hufnagel, 1767)	7	
Psychidae	*Bacotia*	*Bacotia claustrella*	semi-concealed	(Bruand, 1845)	29	h12
Nolidae	*Bena*	*Bena bicolorana*	exposed	(Fuessly, 1775)	28	
Geometridae	*Biston*	*Biston betularia*	exposed	(Linnaeus, 1758)	2	
Geometridae	*Biston*	*Biston strataria*	exposed	(Hufnagel, 1767)	1	h13
Bucculatricidae	*Bucculatrix*	*Bucculatrix thoracella*	both	(Thunberg, 1794)	567	h14
Bucculatricidae	*Bucculatrix*	*Bucculatrix ulmella*	both	Zeller, 1848	36	h15
Tenthredinidae	*Caliroa*	*Caliroa varipes*	exposed	(Klug, 1814)	1	h16
Lymantriidae	*Calliteara*	*Calliteara pudibunda*	exposed	(Linnaeus, 1758)	11	
Gracillariidae	*Caloptilia*	*Caloptilia hemidactylella*	semi-concealed	(Denis & Schiffermüller, 1775)	7	
Gracillariidae	*Caloptilia*	*Caloptilia semifascia*	semi-concealed	(Haworth, 1828)	8	h17
Geometridae	*Campaea*	*Campaea margaritaria*	exposed	(Linnaeus, 1767)	86	h18
Tortricidae	*Capua*	*Capua vulgana*	semi-concealed	(Frölich, 1828)	1	h19
Oecophoridae	*Carcina*	*Carcina quercana*	semi-concealed	(Fabricius, 1775)	35	h20
Noctuidae	*Catocala*	*Catocala promissa*	exposed	(Denis & Schiffermüller, 1775)	2	
Noctuidae	*Catocala*	*Catocala sponsa*	exposed	(Linnaeus, 1767)	16	
Tortricidae	*Clepsis*	*Clepsis consimilana*	semi-concealed	(Hübner, 1817)	1	

Coleophoridae	*Coleophora*	*Coleophora coracipennella*	semi-concealed	(Hübner, 1796)	1	
Coleophoridae	*Coleophora*	*Coleophora flavipennella*	semi-concealed	(Duponchel, 1843)	27	h21
Coleophoridae	*Coleophora*	*Coleophora kuehnella*	semi-concealed	(Goeze, 1783)	4	
Coleophoridae	*Coleophora*	*Coleophora serratella*	semi-concealed	(Linnaeus, 1761)	1	
Noctuidae	*Colocasia*	*Colocasia coryli*	exposed	(Linnaeus, 1758)	6	
Geometridae	*Colotois*	*Colotois pennaria*	exposed	(Linnaeus, 1761)	178	h22
Noctuidae	*Conistra*	*Conistra rubiginosa*	exposed	(Scopoli, 1763)	36	h23
Noctuidae	*Conistra*	*Conistra vaccinii*	exposed	(Linnaeus, 1761)	15	h24
Pyralidae	*Conobathra*	*Conobathra repandana*	semi-concealed	(Fabricius, 1798)	16	h25
Pyralidae	*Conobathra*	*Conobathra tumidana*	semi-concealed	(Denis & Schiffermüller, 1775)	4	h26
Noctuidae	*Cosmia*	*Cosmia affinis*	exposed	(Linnaeus, 1767)	4	
Noctuidae	*Cosmia*	*Cosmia trapezina*	exposed	(Linnaeus, 1758)	150	h27
Noctuidae	*Craniophora*	*Craniophora ligustri*	exposed	(Denis & Schiffermüller, 1775)	25	h28
Geometridae	*Cyclophora*	*Cyclophora annularia*	exposed	(Fabricius, 1775)	101	h29
Geometridae	*Cyclophora*	*Cyclophora punctaria*	exposed	(Linnaeus, 1758)	40	
Geometridae	*Cyclophora*	*Cyclophora ruficiliaria*	exposed	(Herrich-Schäffer, 1855)	42	h30
Drepanidae	*Cymatophorina*	*Cymatophorina diluta*	exposed	(Denis & Schiffermüller, 1775)	1	
Psychidae	*Dahlica*	*Dahlica lichenella*	semi-concealed	(Linnaeus, 1761)	14	
Psychidae	*Dahlica*	*Dahlica triquetrella*	semi-concealed	(Hübner, 1813)	20	
Oecophoridae	*Dasystoma*	*Dasystoma salicella*	semi-concealed	(Hübner, 1796)	10	
Gelechiidae	*Dichomeris*	*Dichomeris ustalella*	semi-concealed	(Fabricius, 1794)	7	
Noctuidae	*Dichonia*	*Dichonia aprilina*	exposed	(Linnaeus, 1758)	1	
Psychidae	*Diplodoma*	*Diplodoma laichartingella*	semi-concealed	(Goeze, 1783)	4	
Oecophoridae	*Diurnea*	*Diurnea fagella*	semi-concealed	(Denis & Schiffermüller, 1775)	53	h31
Oecophoridae	*Diurnea*	*Diurnea lipsiella*	semi-concealed	(Denis & Schiffermüller, 1775)	12	
Notodontidae	*Drymonia*	*Drymonia dodonaea*	exposed	(Denis & Schiffermüller, 1775)	2	
Noctuidae	*Dryobotodes*	*Dryobotodes eremita*	exposed	(Fabricius, 1775)	245	h32
Pyralidae	*Eccopisa*	*Eccopisa effractella*	semi-concealed	Zeller, 1848	1	
Geometridae	*Ectropis*	*Ectropis crepuscularia*	exposed	(Denis & Schiffermüller, 1775)	27	
Arctiidae	*Eilema*	*Eilema sororcula*	exposed	(Hufnagel, 1766)	8	
Pyralidae	*Endotricha*	*Endotricha flammealis*	semi-concealed	(Denis & Schiffermüller, 1775)	1	
Geometridae	*Ennomos*	*Ennomos alniaria*	exposed	(Linnaeus, 1758)	1	
Geometridae	*Ennomos*	*Ennomos autumnaria*	exposed	(Werneburg, 1859)	5	
Geometridae	*Ennomos*	*Ennomos fuscantaria*	exposed	(Haworth, 1809)	14	
Geometridae	*Ennomos*	*Ennomos quercinaria*	exposed	(Hufnagel, 1767)	7	
Tortricidae	*Epinotia*	*Epinotia festivana*	semi-concealed	(Hübner, 1799)	2	
Geometridae	*Epirrita*	*Epirrita dilutata*	exposed	(Denis & Schiffermüller, 1775)	12	h33
Geometridae	*Erannis*	*Erannis defoliaria*	exposed	(Clerck, 1759)	283	h34
Tortricidae	*Eudemis*	*Eudemis porphyrana*	semi-concealed	(Hübner, 1799)	217	
Tortricidae	*Eudemis*	*Eudemis profundana*	semi-concealed	(Denis & Schiffermüller, 1775)	41	
Tenthredinidae	*Eupareophora*	*Eupareophora exarmata*	exposed	(C. G. Thomson, 1871)	10	
Geometridae	*Eupithecia*	*Eupithecia abbreviata*	exposed	Stephens, 1831	6	
Lymantriidae	*Euproctis*	*Euproctis similis*	exposed	(Fuessly, 1775)	13	h35
Noctuidae	*Eupsilia*	*Eupsilia transversa*	exposed	(Hufnagel, 1766)	41	h36
Agonoxenidae	*Haplochrois*	*Haplochrois ochraceella*	semi-concealed	(Rebel, 1903)	1	
Tenthredinidae	*Harpiphorus*	*Harpiphorus lepidus*	exposed	(Klug, 1814)	1	
Tortricidae	*Hedya*	*Hedya nubiferana*	semi-concealed	(Haworth, 1811)	2	
Geometridae	*Hemithea*	*Hemithea aestivaria*	exposed	(Hübner, 1799)	4	
Gelechiidae	*Hypatima*	*Hypatima rhomboidella*	semi-concealed	(Linnaeus, 1758)	1	

Geometridae	*Hypomecis*	*Hypomecis punctinalis*	exposed	(Scopoli, 1763)	14	
Geometridae	*Hypomecis*	*Hypomecis roboraria*	exposed	(Denis & Schiffermüller, 1775)	1	
Geometridae	*Chloroclysta*	*Chloroclysta siterata*	exposed	(Hufnagel, 1767)	29	
Tortricidae	*Choristoneura*	*Choristoneura diversana*	semi-concealed	(Hübner, 1817)	27	h37
Tortricidae	*Choristoneura*	*Choristoneura hebenstreitella*	semi-concealed	(Müller, 1764)	4	
Noctuidae	*Laspeyria*	*Laspeyria flexula*	exposed	(Denis & Schiffermüller, 1775)	5	
Noctuidae	*Lithophane*	*Lithophane ornitopus*	exposed	(Hufnagel, 1766)	21	
Noctuidae	*Lithophane*	*Lithophane semibrunnea*	exposed	(Haworth, 1809)	20	
Arctiidae	*Lithosia*	*Lithosia quadra*	exposed	(Linnaeus, 1758)	28	
Geometridae	*Lycia*	*Lycia hirtaria*	exposed	(Clerck, 1759)	45	h38
Geometridae	*Lycia*	*Lycia pomonaria*	exposed	(Hübner, 1790)	1	
Lymantriidae	*Lymantria*	*Lymantria dispar*	exposed	(Linnaeus, 1758)	135	h39
Lymantriidae	*Lymantria*	*Lymantria monacha*	exposed	(Linnaeus, 1758)	4	
Tenthredinidae	*Macrophya*	*Macrophya punctumalbum*	exposed	(Linnaeus, 1767)	211	
Lasiocampidae	*Malacosoma*	*Malacosoma neustria*	exposed	(Linnaeus, 1758)	50	
Tenthredinidae	*Mesoneura*	*Mesoneura opaca*	exposed	(Fabricius, 1775)	43	
Noctuidae	*Moma*	*Moma alpium*	exposed	(Osbeck, 1778)	2	
Tenthredinidae	*Nematus*	*Nematus umbratus*	exposed	C. G. Thomson, 1871	3	
Lycaenidae	*Neozephyrus*	*Neozephyrus quercus*	exposed	(Linnaeus, 1758)	26	h40
Nolidae	*Nola*	*Nola aerugula*	exposed	(Hübner, 1793)	1	
Nolidae	*Nola*	*Nola cicatricalis*	exposed	(Treitschke, 1835)	1	
Nolidae	*Nola*	*Nola confusalis*	exposed	(Herrich-Schäffer, 1847)	1	
Notodontidae	*Notodonta*	*Notodonta dromedarius*	exposed	(Linnaeus, 1767)	2	
Nolidae	*Nycteola*	*Nycteola revayana*	exposed	(Scopoli, 1772)	2	
Geometridae	*Operophtera*	*Operophtera brumata*	exposed	(Linnaeus, 1758)	3426	h41
Lymantriidae	*Orgyia*	*Orgyia antiqua*	exposed	(Linnaeus, 1758)	36	h42
Lymantriidae	*Orgyia*	*Orgyia recens*	exposed	(Hübner, 1819)	179	
Noctuidae	*Orthosia*	*Orthosia cerasi*	exposed	(Fabricius, 1775)	170	h43
Noctuidae	*Orthosia*	*Orthosia cruda*	exposed	(Denis & Schiffermüller, 1775)	623	h44
Noctuidae	*Orthosia*	*Orthosia gothica*	exposed	(Linnaeus, 1758)	2	
Noctuidae	*Orthosia*	*Orthosia gracilis*	exposed	(Denis & Schiffermüller, 1775)	106	
Noctuidae	*Orthosia*	*Orthosia incerta*	exposed	(Hufnagel, 1766)	12	
Tortricidae	*Pammene*	*Pammene albuginana*	semi-concealed	(Guenée, 1845)	1	
Tortricidae	*Pammene*	*Pammene argyrana*	semi-concealed	(Hübner, 1799)	1	
Tortricidae	*Pammene*	*Pammene fasciana*	semi-concealed	(Linnaeus, 1761)	1	
Tortricidae	*Pammene*	*Pammene giganteana*	semi-concealed	(Peyerimhoff,1863)	12	h45
Tortricidae	*Pammene*	*Pammene splendidulana*	semi-concealed	(Guenée, 1845)	2	
Tortricidae	*Pandemis*	*Pandemis cerasana*	semi-concealed	(Hübner, 1786)	7	h46
Tortricidae	*Pandemis*	*Pandemis corylana*	semi-concealed	(Fabricius, 1794)	16	h47
Tortricidae	*Pandemis*	*Pandemis heparana*	semi-concealed	(Denis & Schiffermüller, 1775)	8	
Geometridae	*Parectropis*	*Parectropis similaria*	exposed	(Hufnagel, 1767)	11	
Geometridae	*Peribatodes*	*Peribatodes rhomboidaria*	exposed	(Denis & Schiffermüller, 1775)	2	
Tenthredinidae	*Periclista*	*Periclista albida*	exposed	(Klug, 1814)	2	
Tenthredinidae	*Periclista*	*Periclista lineolata*	exposed	(Klug, 1814)	3	
Noctuidae	*Perigrapha*	*Perigrapha munda*	exposed	(Denis & Schiffermüller, 1775)	28	h48
Geometridae	*Phigalia*	*Phigalia pilosaria*	exposed	(Denis & Schiffermüller, 1775)	56	h49
Pyralidae	*Phycita*	*Phycita roborella*	semi-concealed	(Denis & Schiffermüller, 1775)	32	
Gracillariidae	*Phyllonorycter*	*Phyllonorycter acerifoliella*	semi-concealed	(Zeller, 1839)	1	
Gracillariidae	*Phyllonorycter*	*Phyllonorycter esperella*	semi-concealed	(Goeze, 1783)	1	

Geometridae	*Plagodis*	*Plagodis dolabraria*	exposed	(Linnaeus, 1767)	4	
Lasiocampidae	*Poecilocampa*	*Poecilocampa populi*	exposed	(Linnaeus, 1758)	1	
Tenthredinidae	*Pristiphora*	*Pristiphora leucopus*	exposed	(Hellén, 1948)	8	
Tenthredinidae	*Pristiphora*	*Pristiphora subbifida*	exposed	(Thomson, 1871)	3	
Psychidae	*Proutia*	*Proutia betulina*	semi-concealed	(Zeller, 1839)	20	
Nolidae	*Pseudoips*	*Pseudoips prasinana*	exposed	(Linnaeus, 1758)	28	
Gelechiidae	*Psoricoptera*	*Psoricoptera gibbosella*	semi-concealed	(Zeller, 1839)	4	
Psychidae	*Psyche*	*Psyche casta*	semi-concealed	(Pallas, 1767)	11	h50
Notodontidae	*Ptilodon*	*Ptilodon capucina*	exposed	(Linnaeus, 1758)	5	
Notodontidae	*Ptilodon*	*Ptilodon cucullina*	exposed	(Denis & Schiffermüller, 1775)	3	
Notodontidae	*Ptilophora*	*Ptilophora plumigera*	exposed	(Denis & Schiffermüller, 1775)	186	h51
Tortricidae	*Ptycholoma*	*Ptycholoma lecheana*	semi-concealed	(Linnaeus, 1758)	2	
Tenthredinidae	*Rhogogaster*	*Rhogogaster chlorosoma*	exposed	(Benson, 1943)	3	
Roeslerstammiidae	*Roeslerstammia*	*Roeslerstammia erxlebella*	exposed	(Fabricius, 1787)	12	
Roeslerstammiidae	*Roeslerstammia*	*Roeslerstammia pronubella*	exposed	(Denis & Schiffermüller, 1775)	1	
Noctuidae	*Scotochrosta*	*Scotochrosta pulla*	exposed	(Denis & Schiffermüller, 1775)	1	
Geometridae	*Selenia*	*Selenia tetralunaria*	exposed	(Hufnagel, 1767)	14	
Notodontidae	*Spatalia*	*Spatalia argentina*	exposed	(Denis & Schiffermüller, 1775)	2	
Tortricidae	*Spilonota*	*Spilonota ocellana*	semi-concealed	(Denis & Schiffermüller, 1775)	3	
Gelechiidae	*Stenolechia*	*Stenolechia gemmella*	semi-concealed	(Linnaeus, 1758)	1	
Argidae	*Sterictiphora*	*Sterictiphora longicornis*	both	Chevin, 1982	6	
Psychidae	*Sterrhopterix*	*Sterrhopterix fusca*	semi-concealed	(Haworth, 1809)	8	
Psychidae	*Taleporia*	*Taleporia tubulosa*	semi-concealed	(Retzius, 1783)	16	
Gelechiidae	*Teleiodes*	*Teleiodes luculella*	semi-concealed	(Hübner, 1813)	1	
Tenthredinidae	*Tomostethus*	*Tomostethus nigritus*	exposed	(Fabricius, 1804)	137	h52
Tortricidae	*Tortricodes*	*Tortricodes alternella*	semi-concealed	(Denis & Schiffermüller, 1775)	76	h53
Tortricidae	*Tortrix*	*Tortrix viridana*	semi-concealed	Linnaeus, 1758	35	h54
Drepanidae	*Watsonalla*	*Watsonalla binaria*	exposed	(Hufnagel, 1767)	8	
Ypsolophidae	*Ypsolopha*	*Ypsolopha alpella*	semi-concealed	(Denis & Schiffermüller, 1775)	5	
Ypsolophidae	*Ypsolopha*	*Ypsolopha lucella*	semi-concealed	(Fabricius, 1775)	6	h55
Ypsolophidae	*Ypsolopha*	*Ypsolopha parenthesella*	semi-concealed	(Linnaeus, 1761)	4	
Ypsolophidae	*Ypsolopha*	*Ypsolopha sequella*	semi-concealed	(Clerck, 1759)	10	h56
Ypsolophidae	*Ypsolopha*	*Ypsolopha sylvella*	semi-concealed	(Linnaeus, 1767)	2	
Ypsolophidae	*Ypsolopha*	*Ypsolopha ustella*	semi-concealed	(Clerck, 1759)	25	h57
Tortricidae	*Zeiraphera*	*Zeiraphera isertana*	semi-concealed	(Fabricius, 1794)	119	
Bucculatricidae	*Bucculatrix*	unidentified	both		18	
Tortricidae	*Acleris*	unidentified	semi-concealed		3	
Pyralidae	*Conobathra*	unidentified	semi-concealed		3	
Geometridae	*Agriopis*	unidentified	exposed		4	
Geometridae	*Alsophila*	unidentified	exposed		1	
Oecophoridae	*Carcina*	unidentified	semi-concealed		1	
Coleophoridae	*Coleophora*	unidentified	semi-concealed		5	
Tortricidae	*Eudemis*	unidentified	semi-concealed		2	
Geometridae	*Eupithecia*	unidentified	exposed		2	
Tortricidae	*Choristoneura*	unidentified	semi-concealed		1	
Noctuidae	*Orthosia*	unidentified	exposed		2	
Tortricidae	*Pammene*	unidentified	semi-concealed		5	
Pamphiliidae	*Pamphilius*	unidentified	semi-concealed		5	
Tortricidae	*Pandemis*	unidentified	semi-concealed		3	

Gelechiidae	*Psoricoptera*	unidentified	semi-concealed	1
Lymantriidae	*Euproctis*	unidentified	exposed	1
Ypsolophidae	*Ypsolopha*	unidentified	semi-concealed	4
Arctiidae	unidentified	unidentified	exposed	5
Coleophoridae	unidentified	unidentified	semi-concealed	1
Geometridae	unidentified	unidentified	exposed	239
Gracillariidae	unidentified	unidentified	semi-concealed	1
Noctuidae	unidentified	unidentified	exposed	4
Pyralidae	unidentified	unidentified	semi-concealed	2
Tenthredinidae	unidentified	unidentified	exposed	53
Tortricidae	unidentified	unidentified	semi-concealed	19
unidentified	unidentified	unidentified	exposed	3
unidentified	unidentified	unidentified	NA	125

*Semi-concealed feeders included also 58 individuals of leaf miners (Coleophoridae, Gracillariidae, Elachistidae = Agonoxenidae), which fed externally on provided leaves in the laboratory.

References:

Lacourt, J. (1999) Répertoire des Tenthredinidae ouest-paléarctiques (Hymenoptera, Symphyta). Société entomologique de France.

Laštůvka, Z. & Liška, J. (2010) Checklist of Lepidoptera of the Czech Republic (Insecta: Lepidoptera). URL http://lepidoptera.wz.cz.
