# Supplementary material for: Vertical canopy gradient shaping the stratification of leaf‐chewer–parasitoid interactions in a temperate forest
Source: Ecol Evol. 2018 Jun 27;8(15):7297–311. doi: 10.1002/ece3.4194 (PMC6106176; doi:10.1002/ece3.4194)
Supplement: Supplementary file 9 [file ECE3-8-7297-s009.pdf]

Table S4. Pairwise comparison of species dispersion (variance) among tree species. Permutation test of multivariate homogeneity of group dispersion was used to test whether one or more groups is more variable than the others. Non-significant values are abbreviated as n.s.

	Acer campestre	*Carpinus betulus*	*Fraxinus* spp.	*Quercus cerris*	*Quercus robur*	*Tilia cordata*	*Ulmus laevis*
Acer campestre	NA	n.s.	$p = 0.037$	n.s.	$p = 0.033$	$p = 0.035$	n.s.
Carpinus betulus	n.s.	NA	$p < 0.01$	$p < 0.01$	$p < 0.01$	$p < 0.01$	n.s.
Fraxinus spp.	$p = 0.031$	$p < 0.01$	NA	n.s.	n.s.	n.s.	$p = 0.039$
Quercus cerris	n.s.	$p = 0.01$	n.s.	NA	n.s.	n.s.	n.s.
Quercus robur	$p = 0.036$	$p < 0.01$	n.s.	n.s.	NA	n.s.	n.s.
Tilia cordata	$p = 0.032$	$p < 0.001$	n.s.	n.s.	n.s.	NA	$p = 0.012$
Ulmus laevis	n.s.	n.s.	$p = 0.048$	n.s.	n.s.	$p = 0.015$	NA
