# Supplementary material for: Vertical canopy gradient shaping the stratification of leaf‐chewer–parasitoid interactions in a temperate forest
Source: Ecol Evol. 2018 Jun 27;8(15):7297–311. doi: 10.1002/ece3.4194 (PMC6106176; doi:10.1002/ece3.4194)
Supplement: Supplementary file 10 [file ECE3-8-7297-s010.pdf]

Table S5. Mean values of all quantitative network indices and community metrics for individual tree species and canopy levels.

Tree species and canopy level	Host species diversity	Parasitoid species diversity	Interaction events (Matrix size)	H_2'	Generality	Vulnerability	Interaction evenness	Connectance	Linkage density	NODF	Number of compartments	Modularity
Acer campestre												
first	3.500	6.500	17.250	1.000	1.000	2.034	0.324	0.094	1.517	0.000	3.500	0.481
second	6.500	11.250	23.500	0.960	1.111	2.651	0.434	0.086	1.881	0.872	5.000	0.630
third	4.250	5.000	9.750	0.991	1.048	1.279	0.303	0.072	1.163	0.000	3.750	0.579
total	4.750	7.583	16.833	0.984	1.053	1.988	0.354	0.084	1.520	0.291	4.083	0.571
Carpinus betulus												
first	3.000	4.000	4.333	1.000	1.000	1.556	0.332	0.073	1.278	0.000	3.000	0.574
second	4.000	6.333	7.000	0.955	1.095	2.275	0.379	0.066	1.685	0.000	3.667	0.576
third	3.667	6.000	7.333	1.000	1.000	1.860	0.321	0.058	1.430	0.000	3.667	0.638
total	3.556	5.444	6.222	0.985	1.032	1.897	0.344	0.066	1.464	0.000	3.444	0.591
Fraxinus spp.												
first	4.000	4.500	8.500	0.885	1.483	1.412	0.369	0.086	1.448	1.724	2.000	0.528
second	3.000	4.500	5.500	0.829	1.191	2.246	0.360	0.077	1.718	0.909	2.500	0.476
third	2.500	3.500	5.000	0.772	1.333	2.000	0.390	0.119	1.667	0.000	2.000	0.354
total	3.167	4.167	6.333	0.829	1.336	1.886	0.373	0.094	1.611	0.878	2.167	0.453
Quercus cerris												
first	7.000	11.000	33.000	0.901	1.233	2.286	0.424	0.062	1.760	0.885	4.333	0.674
second	8.667	14.333	84.333	0.821	1.377	2.691	0.397	0.058	2.034	1.429	4.667	0.557
third	5.667	8.000	19.000	0.919	1.278	1.677	0.368	0.061	1.477	1.219	4.000	0.619
total	7.111	11.111	45.444	0.880	1.296	2.218	0.396	0.060	1.757	1.177	4.333	0.617
Quercus robur												
first	5.000	5.000	5.000	1.000	1.000	1.000	0.349	0.040	1.000	0.000	5.000	0.800
second	3.000	4.000	4.000	1.000	1.000	1.500	0.310	0.048	1.250	0.000	3.000	0.625
third	2.000	2.000	2.000	1.000	1.000	1.000	0.175	0.036	1.000	0.000	2.000	0.500
total	3.333	3.667	3.667	1.000	1.000	1.167	0.278	0.041	1.083	0.000	3.333	0.642
Ulmus laevis												
first	4.000	7.000	12.000	1.000	1.000	2.000	0.366	0.083	1.500	0.000	4.000	0.625
second	2.000	5.000	7.000	1.000	1.000	2.419	0.347	0.090	1.709	0.000	2.000	NA
third	5.000	8.000	9.000	0.895	1.222	2.333	0.459	0.077	1.778	0.000	4.000	0.716
total	3.667	6.667	9.333	0.965	1.074	2.251	0.391	0.084	1.662	0.000	3.333	0.671
All trees												
first	4.357	6.571	15.357	0.962	1.119	1.820	0.358	0.077	1.470	0.436	3.500	0.590
second	5.357	8.929	27.857	0.916	1.160	2.422	0.389	0.072	1.791	0.685	3.929	0.577
third	4.071	5.643	9.929	0.940	1.137	1.647	0.336	0.071	1.392	0.261	3.429	0.567
total	4.595	7.048	17.714	0.939	1.139	1.963	0.361	0.073	1.551	0.461	3.619	0.578
