# Supplementary material for: Vertical canopy gradient shaping the stratification of leaf‐chewer–parasitoid interactions in a temperate forest
Source: Ecol Evol. 2018 Jun 27;8(15):7297–311. doi: 10.1002/ece3.4194 (PMC6106176; doi:10.1002/ece3.4194)
Supplement: Supplementary file 11 [file ECE3-8-7297-s011.pdf]

Table S6. Results of likelihood-ratio (LR) analysis of deviance testing the partial effect of explanatory variables and its interactions on the values of the food web metrics. LR tested whether the deviance of the full model significantly increases after removing each explanatory variable from the model. Chi-square (ΔG) shows the change in deviance due to each term in the model. Significant p-values are in bold.

	Df	Deviance	AIC	ΔG	P(Chisq)
**Linkage density**					
<full model>		0.592	-39.800		
Canopy level	2	0.709	-36.182	7.617	**0.022**
Tree species	5	0.796	-37.373	12.427	**0.029**
Tree height	1	0.605	-40.888	0.912	0.339
<full model>		0.324	-37.098		
Canopy level: Tree species	10	0.452	-43.128	13.971	0.174
Canopy level: Tree height	2	0.333	-39.973	1.124	0.569
Tree species: Tree height	2	0.441	-28.176	12.922	**<0.01**
**Interaction evenness**					
<full model>		4.234	42.824		
Canopy level	2	4.492	41.305	2.481	0.289
Tree species	5	4.731	37.484	4.661	0.458
Tree height	1	4.265	41.135	0.311	0.576
<full model>		2.159	42.551		
Canopy level: Tree species	10	3.571	43.671	21.119	**0.021**
Canopy level: Tree height	2	2.581	46.021	7.468	**0.023**
Tree species: Tree height	2	2.493	44.586	6.034	**0.048**
**H2**					
<full model>		0.412	-55.984		
Canopy level	2	0.417	-58.498	1.485	0.475
Tree species	5	0.576	-50.915	15.069	**0.011**
Tree height	1	0.407	-57.497	0.487	0.485
<full model>		0.116	-79.918		
Canopy level: Tree species	10	0.158	-87.189	12.729	0.239
Canopy level: Tree height	2	0.124	-81.351	2.567	0.277
Tree species: Tree height	2	0.355	-37.251	46.667	**<0.001**
**Generality HL**					
<full model>		0.475	-48.991		
Canopy level	2	0.486	-52.049	0.941	0.624
Tree species	5	0.645	-46.148	12.842	**0.024**
Tree height	1	0.481	-50.486	0.505	0.477
<full model>		0.156	-67.782		
Canopy level: Tree species	10	0.235	-70.586	17.196	0.071
Canopy level: Tree height	2	0.175	-66.847	4.935	0.084
Tree species: Tree height	2	0.393	-32.927	38.855	**<0.001**
**Vulnerability LL**					
<full model>		1.105	-13.578		
Canopy level	2	1.389	-7.981	9.597	**<0.01**
Tree species	5	1.385	-14.088	9.491	0.091
Tree height	1	1.146	-14.041	1.536	0.215
<full model>		0.707	-4.313		
Canopy level: Tree species	10	1.002	-9.697	14.625	0.146
Canopy level: Tree height	2	0.731	-6.960	1.352	0.508
Tree species: Tree height	2	0.769	-4.821	3.491	0.174
**Weighted Connectance**					
<full model>		44.290	141.42		
Canopy level	2	45.469	138.52	1.102	0.576
Tree species	5	73.346	152.61	21.186	**<0.001**
Tree height	1	48.740	143.44	4.021	**0.044**
<full model>		35.260	159.84		
Canopy level: Tree species	10	39.611	144.73	4.886	0.898
Canopy level: Tree height	2	35.580	156.22	0.379	0.827
Tree species: Tree height	2	37.901	158.88	3.033	0.219

**Modularity**					
<full model>		3.299	33.568		
Canopy level	2	3.398	30.661	1.092	0.579
Tree species	4	4.302	35.390	9.821	**0.043**
Tree height	1	3.577	34.555	2.986	0.083
<full model>		2.288	44.031		
Canopy level: Tree species	8	2.751	34.840	6.809	0.557
Canopy level: Tree height	2	2.377	41.438	1.406	0.495
Tree species: Tree height	2	2.555	44.117	4.085	0.129
**Weighted NODF**					
<full model>		2.666	23.406		
Canopy level	2	2.681	19.635	0.229	0.891
Tree species	5	3.214	21.254	7.847	0.164
Tree height	1	2.686	21.716	0.310	0.577
<full model>		1.380	23.749		
Canopy level: Tree species	10	2.308	25.339	21.591	**0.017**
Canopy level: Tree height	2	1.771	30.219	10.469	**<0.01**
Tree species: Tree height	2	1.718	28.947	9.198	**0.011**
**Number of compartments**					
<full model>		11.186	83.624		
Canopy level	2	11.266	79.924	0.301	0.861
Tree species	5	16.029	88.733	15.108	**<0.01**
Tree height	1	13.583	89.778	8.153	**<0.01**
<full model>		7.298	93.691		
Canopy level: Tree species	10	9.677	85.541	11.849	0.295
Canopy level: Tree height	2	7.559	91.164	1.472	0.478
Tree species: Tree height	2	8.331	95.247	5.555	0.062
